# Heterogeneity in Risk-Taking During the COVID-19 Pandemic: Evidence From the UK Lockdown

**DOI:** 10.3389/fpsyg.2021.643653

**Published:** 2021-04-01

**Authors:** Benno Guenther, Matteo M. Galizzi, Jet G. Sanders

**Affiliations:** ^1^Department of Psychological and Behavioural Science, London School of Economics and Political Science, London, United Kingdom; ^2^Salient Behavioural Consultants Ltd., London, United Kingdom

**Keywords:** risk taking, risk preferences, risk tolerance, risk compensation, COVID-19, lockdown

## Abstract

In two pre-registered online studies during the COVID-19 pandemic and the early 2020 lockdown (one of which with a UK representative sample) we elicit risk-tolerance for 1,254 UK residents using four of the most widely applied risk-taking tasks in behavioral economics and psychology. Specifically, participants completed the incentive-compatible Balloon Analog Risk Task (BART) and the Binswanger-Eckel-Grossman (BEG) multiple lotteries task, as well as the Domain-Specific Risk-Taking Task (DOSPERT) and the self-reported questions for risk-taking used in the German Socio-economic Panel (SOEP) study. In addition, participants in the UK representative sample answered a range of questions about COVID-19-related risky behaviors selected from the UCL COVID-19 Social Survey and the ICL-YouGov survey on COVID-19 behaviors. Consistently with pre-COVID-19 times, we find that risk tolerance during the UK lockdown (i) was higher in men than in women and (ii) decreased with age. Undocumented in pre-COVID-19 times, we find some evidence for healthier participants displaying significantly higher risk-tolerance for self-reported risk measures. We find no systematic nor robust patterns of association between the COVID-19 risky behaviors and the four risk-taking tasks in our study. Moreover, we find no evidence in support of the so-called “risk compensation” hypothesis. If anything, it appears that participants who took greater risk in real-life COVID-19-relevant risky behaviors (e.g., isolating or taking precautions) also exhibited higher risk-tolerance in our experimental and self-reported risk-taking measures.

## Introduction

There is increasing evidence in behavioral economics and psychology of high heterogeneity in risk tolerance across individuals, groups, and populations (e.g., Vieider et al., 2015a,b; Falk et al., 2018). Risk tolerance—as broadly captured by individual risk preferences, risk attitudes, and risk perceptions—is considered a fundamental driver of individual behavior in a broad array of contexts, including health, social care, education, migration, occupational and self-employment choices, personal, and household finance (Barsky et al., 1997; Gollier, 2001; Bonin et al., 2007; Anderson and Mellor, 2008; Bellemare and Shearer, 2013; Sutter et al., 2013; Sanders and Jenkins, 2016).

To date, very little is known on the heterogeneity in risk tolerance over the course of the current COVID-19 pandemic. This study aims at contributing toward filling this gap.

To this effect, we look at inter-individual differences in risk-taking during the first lockdown period in the UK (23 March 2020–26 May 2020) as a consequence of the rapid spread of the COVID-19 pandemic. Specifically, we elicit risk tolerance using four of the most widely applied risk-taking tasks in behavioral economics and psychology in two pre-registered online studies, one of which with a UK representative sample. Participants completed the incentive-compatible Balloon Analog Risk Task (BART; Lejuez et al., 2002) and the incentive-compatible Binswanger-Eckel-Grossman (BEG) multiple lotteries task (Binswanger, 1980; Eckel and Grossman, 2002). They also answered the Domain-Specific Risk-Taking Task (DOSPERT; Weber et al., 2002) and the self-reported questions for risk-taking from the German Socio-economic panel (SOEP) study (Wagner et al., 2011). Our reason for using multiple measures of risk taking, both incentive-compatible and self-reported, is that it remains unclear whether, and to which extent, the different measures relate to one another, and whether, and to which extent they tap into different dimensions of human risk taking behavior in the real world (Barseghyan et al., 2011; Einav et al., 2012; Loomes and Pogrebna, 2014; Crosetto and Filippin, 2016; Galizzi et al., 2016a,b; Sanders and Jenkins, 2016; Frey et al., 2017; Menkhoff and Sakha, 2017; Pedroni et al., 2017; Brañas-Garza et al., 2018; Charness et al., 2020).

We use our data to address three main research questions. First, we systematically investigate heterogeneity in risk tolerance by gender (male, female), age group (separated by median age), and pre-existing health status (separated by self-reported median health score). We look at age and gender specifically to verify two most commonly reported findings in the risk-taking literature before the COVID-19 pandemic, namely that men and younger respondents take more risks. Although no systematic relationship between health status and risk tolerance has previously been established (e.g., Galizzi and Miraldo, 2017), under the COVID-19 pandemic health status has been shown to be a significant determinant of the likelihood of suffering from severe consequences, of being hospitalized, and of dying (e.g., Richardson et al., 2020). We therefore test for this association as well in our data.

Second, we look at the relationship between (i) our experimental and self-reported risk-taking measures and (ii) self-reported real-world behaviors deemed to be of high risk at all times (e.g., drinking, smoking) and of particular relevance during the COVID-19 pandemic (e.g., hand washing, self-isolation, mask wearing). Understanding these relationships between risk measures and behaviors under the current pandemic circumstances has three purposes. First, it allows us to observe whether risk measures under the current times are still predicting real-world behaviors they have previously been linked to. Second, it allows us to establish the predictive relationship between risk measures and COVID-19-related risky behaviors. Third, it allows us to map different relationships between risk measures and behaviors across segments of the population, as this can be of use for identifying potential routes of behavioral intervention in the current crisis. For example, certain subgroups of the population are more susceptible to developing severe symptoms in reaction to the virus (Pérez-López et al., 2020; Zhang et al., 2020). This may influence their perception of the virus and their behavior in response: for example, such segments of the population may be less willing to take daily risks in order to reduce their chance of exposure to the virus and, in turn, be more responsive to interventions which leverage perceived risk.

Third, we explore whether there is evidence in our data for the so-called “risk compensation” hypothesis. The risk compensation or “risk homeostasis” hypothesis suggests that people typically adjust their behavior in response to changes in (perceived) levels of risk, and that this occurs in a compensatory way. As a common example, wearing a seat belt could potentially lead to an increase in speeding (e.g., Peltzman, 1975; Houston and Richardson, 2007).

In the context of COVID-19, we could think of similar examples: wearing a mask (and the associated reduction in perceived risk) could lead to reduced social distancing, as suggested by a recent online experiment by Luckman et al. (2020); or (from studies in pre-COVID-19 times) getting vaccinated could result in increased carelessness, as found by some (e.g., Brewer et al., 2007; Eaton and Kalichman, 2007), but not by others (Kasting et al., 2016; Madhivanan et al., 2016).

More specifically, we explore two distinct potential behavioral channels behind risk compensation. One channel would consist in potential compensation among risk taking activities occurring within the same health-related domain: taking less health-related risks in the context of COVID-19 protective behavior (e.g., increased mask wearing) could potentially lead to taking more risks in other COVID-19 or health-related behaviors (e.g., reduced social distancing). This is theoretically plausible and would also be compatible with the idea that people engage in risk taking across overarching motives or life accounts (“being healthy”) rather than narrowly defined specific behaviors (“drinking”).

The other channel is arguably more speculative: it would predict that risk-taking in the health-related domain could lead to compensatory (or reinforcing) risk-taking in another domain. There is a literature providing some evidence in support of such a channel in relation to the broader distinction in behavioral economics between “background” risk and “foreground” risk (Eeckhoudt et al., 1996; Guiso and Paiella, 2008; Noussair et al., 2014). This literature typically operationalizes this distinction by looking at how an exogenous shock in the background risk due, for example, to a natural disaster or a terrorist attack, leads to more or less risk taking into a foreground risk-taking behavior such as for example playing a lottery or an experimental risk-taking game in the field. Interestingly, the evidence provided by this literature is really mixed, with some studies finding evidence of less risk taking in playing lotteries or experimental games after a natural disaster (Charness et al., 2013; Cameron and Shah, 2015), while some other studies finding evidence of more risk taking in those lotteries or games after a natural disaster (Eckel et al., 2009; Bchir and Willinger, 2013; Page et al., 2014; Said et al., 2015). In other words, the available evidence on this channel supports not only the risk compensation hypothesis but also the alternative “diminished sensitivity” hypothesis (see more below).

The theoretical and conceptual framework for this second channel to plausibly occur and take place is far less clear than for the former channel. For example, the behavioral mechanisms behind the latter channel do not seem consistent with the idea that risk-taking is highly domain-specific. Evidence is growing, for example, that risk-taking can significantly differ across different domains (such as health and finance), across different areas of insurance behavior, or between self-reported and experimental risk-taking tasks (e.g., Barseghyan et al., 2011; Einav et al., 2012; Riddel, 2012; Galizzi et al., 2016a; Charness et al., 2020). Also, the well-documented “mental accounting” phenomenon in behavioral economics would suggest that people tend to think in terms of separate life “accounts” (e.g., savings, consumption, health), which would also point against the hypothesis that spillovers in risk taking exist across different risk taking domains.

In sum, if there were to be evidence for the risk compensation hypothesis, we should be able to observe this in our data through evidence from the first channel: that respondents who take less COVID-19-related risks in the real world (e.g., by wearing a mask) take *more* risks in other real-world risky health behaviors (e.g., less isolation or hand washing), and vice versa. For the sake of completeness and transparency however, we also report the empirical results related to the second possible channel in Supplementary Material A, namely the empirical test of the conjecture that respondents who take less COVID-19-related risks in the real world (e.g., by wearing a mask) take more risks in the risk-taking tasks in our online experiment.

## Background and Related Literature

### Heterogeneity in Risk Tolerance

A number of studies have looked into the heterogeneity of risk preferences (e.g., Hey and Orme, 1994; Starmer, 2000; Andersen et al., 2008, 2010; Harrison and Rutström, 2009; Bruhin et al., 2010; Wakker, 2010; Dohmen et al., 2011; Balcombe and Fraser, 2015; Vieider et al., 2015a,b; Falk et al., 2018). While a systematic review is beyond the scope of the current paper, we briefly summarize some of the most robust findings about heterogeneity in risk tolerance prior to the COVID-19 pandemic, namely differences based on gender and age.

#### Gender

In a large-scale study conducted by Falk et al. (2018) heterogeneity in preferences was investigated among 80,000 participants across 76 countries using a combination of a lottery choice sequence (Falk et al., 2016) and the self-reported general SOEP risk-taking question. Amongst their most robust findings, women displayed substantially less risk tolerance than men. This is a result that has also been found using other risk tasks (Investment Game: Charness and Gneezy, 2012) and other (representative) samples (Dohmen et al., 2011).

Byrnes et al. (1999) conducted a meta-analysis of a total of 150 studies looking at this relationship across a wide range of risk-taking measures and real-life activities. They found that men were more risk-taking across most categories (they did not find a significant effect for framing tasks, Tversky and Kahneman, 1981). They also noted, however, that the effect tended to be relatively small and was often not significant. Croson and Gneezy (2009) reviewed studies on gender differences in preferences (including risk-taking) and concluded that men were more risk taking than women.

It is important to note that not all studies find gender-related differences in risk tolerance. In fact, approximately 40% of the studies do not (Niederle, 2014). Niederle (2014) noted that the mixed evidence could potentially be attributed to small sample sizes. Filippin and Crosetto (2016) surveyed the experimental literature in the context of the Holt and Laury (2002) binary lottery task around gender differences in risk-taking and concluded gender differences to be highly dependent on the “features” of the elicitation method used.

In a study with a total of 2,939 students across 30 countries, Vieider et al. (2015b) elicit risk preferences using certainty equivalents for 44 incentive compatible choice questions. Moreover, participants self-reported their willingness to take risk for the general as well as the domain specific SOEP questions. They found that male students were more risk prone than the females in the study. Yet, they distinguished between risk taking in gains and losses and found that the difference was only significant for gains, but not losses. They also noted that there were no significant gender effects for the health or social domain. This study was consistent with earlier findings by Schubert et al. (1999) who asked participants to make risky choices framed either in terms of investment (gain) and insurance (loss) decisions (treatment condition), or in terms of identical but neutrally framed gambling choices (control condition). They found that women were more risk-seeking compared to men in the situation where a gamble was framed as a loss as opposed to a gain. While in sum males appear to display higher risk preference, this finding seems to be related to situations where sensitivity to gains (vs. losses) is important.

#### Age

Falk et al. (2018) also provided robust evidence for heterogeneity in risk preference between age groups. They showed using the lottery choice sequence (Falk et al., 2016) and the self-reported general SOEP risk-taking question that older participants displayed substantially less risk tolerance than younger participants. This too has been demonstrated with other risk tasks (e.g., BART; Rolison et al., 2012) and samples (e.g., Dohmen et al., 2011).

Vieider et al. (2015b) found weak (and inconsistent) effects for age which they attributed to the narrow age range of their student participants. Dohmen et al. (2017) analyzed the data of representative Dutch and German panel data sets and found that self-reported willingness to take risks decreased with age.

In a meta-analysis, Mata et al. (2011) reviewed 29 studies investigating age-related differences in risk-taking across a range of different tasks, including the Iowa Gambling Task (IGT; Bechara et al., 1994), the behavioral investment allocation strategy (BIAS; Kuhnen and Knutson, 2005), and the BART amongst others. They found older participants to be more risk seeking than younger participants in the IGT as well as the BIAS, but younger participants to be more risk seeking than older participants in the BART. They concluded that age-related differences are highly dependent on the characteristics of the task, in particular their respective learning requirements.

### Risk Tolerance Since the Start of the COVID-19 Pandemic

Few recent studies have investigated risk tolerance and heterogeneity in risk-taking since the start of the COVID-19 pandemic. We briefly summarize their findings below.

Most studies asked the question: is risk tolerance different in times of COVID-19? The answers to this question were mixed.

Arguably the most comprehensive and nuanced analysis comes from Harrison et al. (2020), who elicit atemporal risk preferences, time preferences, and inter-temporal risk preferences (as well as subjective beliefs) in a multi-wave online experiment with 598 undergraduate students in Atlanta, US, between May and October 2020. They model heterogeneity in risk preferences by structurally estimating risk preferences under both the expected utility theory and the rank dependent utility theory frameworks, the latter using a flexible two-parameter probability weighting function. They found that atemporal risk preferences during the COVID-19 pandemic changed significantly compared to before the pandemic. In particular, while before the pandemic respondents were roughly risk neutral, after the pandemic they appeared to be overall risk averse. Harrison et al. (2020) showed, however, that it is important to correctly identify the underlying structure of those preferences. Their results, in fact, are due to the identification of a shift from a “global probability optimism” to a “local probability optimism *and* local probability pessimism” risk perception within the rank dependent utility framework: while before the pandemic respondents were putting greater probability weights on the better lottery prizes, after the pandemic they were placing greater probability weights on the extreme outcomes, using an inverse-S probability weighting function. Together with a concave utility function, this shift in the probability weighting function explained the estimated positive risk premium. Harrison et al. (2020) discussed how the effect of background risk thus crucially depends on the nature of probability weighting, and relate their findings to the phenomena of “risk vulnerability” (i.e., small increases in background risk presumably leading to more risk aversion in the foreground risky choices) and “diminished sensitivity” (i.e., for an individual who is already at a point of sufficiently high background risk, the addition of a small amount of foreground risk will not be particularly salient).

Another detailed structural analysis is the one by Drichoutis and Nayga (2020) who measured risk preferences in a sample of 1,008 undergraduate students in Athens before (February to mid-March in 2019 and 2020) and after the first wave of the pandemic in Greece (late March to May) using the general SOEP question, the Holt and Laury (2002) multiple price list (MPL) measure, and a 15-item version of the DOSPERT (Drichoutis and Vassilopoulos, 2016). They structurally estimated risk preferences and found no statistically significant differences in the sample estimates of the risk preferences before and after the first wave of the pandemic.

Similarly, Angrisani et al. (2020) measure risk taking in a sample of 60 undergraduate students and 48 professional traders in London before and after the first wave of the pandemic in the UK using the Bomb Risk Elicitation Task (BRET) measure (Crosetto and Filippin, 2013), and find no statistically significant changes in the risk preferences in their samples. In seeming contrast, Ikeda et al. (2020) measured risk tolerance in an online survey of 19,737 respondents in Japan between March and June 2020 using hypothetical questions, and found an increase in risk tolerance over time. Aksoy et al. (2021) elicited risk preferences and other economic preferences in a sample of 1,000 MTurk respondents in November–December 2020: they found that their respondents after the pandemic were significantly less risk averse than the respondents in the study by Snowberg and Yariv (2021) before the pandemic.

It is difficult to infer how generalizable these patterns are, because the tasks and statistical methods used to elicit risk preferences, the settings and circumstances of the studies, and the cultural groups are different. Three studies from China may provide some insight into some of the cultural discrepancies observed above. Bu et al. (2020) asked 225 graduate students from the Wuhan University of Science and Technology in China two general and hypothetical questions on risk preferences before and after the COVID-19 pandemic. They found a *decrease* in risk tolerance after the pandemic, and that students who were quarantined in Wuhan displayed higher risk tolerance than students from other cities in the Hubei province or other areas in China. In contrast, Shachat et al. (2020) measured risk preferences of 396 students from Wuhan University using an incentive-compatible lottery between January and March 2020, and found an *increase* in risk tolerance in the early stages of the COVID-19 pandemic. Yet another group (Lohmann et al., 2020) measured risk preferences of 539 students from Beijing University using incentive-compatible lotteries and hypothetical tasks and found *no significant changes* in risk tolerance during the outbreak of the pandemic. This suggests that fluctuations in response to the COVID-19 pandemic may have been specific to the contextual circumstances, the type of task, and perhaps the lockdown restrictions which were in place, and the related perceived risks at the time.

### Heterogeneity of Risk-Taking Since the Start of the COVID-19 Pandemic

Given the recency of the emergence of the COVID-19 pandemic, there is a limited number of studies to date that investigated heterogeneity in risk-taking within their sample. Fan et al. (2020), for example, analyzed the data of a weekly panel conducted with a US representative sample of 5,500 participants during April 2020. They found strong evidence for heterogeneity in COVID-19-related precautionary actions, such as self-isolating or washing hands more often. In particular, they found that their female participants were significantly more likely to engage with these protective behaviors relative to their male participants. Considering the background risk of COVID-19, increases in protective behaviors could be interpreted as a sign of increased risk aversion. Consistently, the same panel data, which also included the SOEP general question for risk-taking, showed that women self-reported lower levels of risk-tolerance than men (Fan et al., 2020). While Drichoutis and Nayga (2020) and Chuang and Liu (2020) also found male participants to exhibit higher risk tolerance than their female participants, Angrisani et al. (2020), on the other hand, did not find any effects associated with either gender or age.

Another recent study by Heo et al. (in press) analyzed financial risk tolerance measured using a 13-item scale developed by Grable and Lytton (1999) of 18,193 financial decision makers. They found that participants with higher financial risk tolerance scores were more likely to be men and less likely to be aged between 55 and 74. Yet another study investigating precautionary behavior in Nigeria by Iorfa et al. (2020) found older participants to be more likely to use precautions against COVID-19. However, they did not find evidence for gender-related differences in precautionary behavior.

As can be seen, the literature to date has employed a broad variety of methods to measure risk tolerance, both incentive-compatible and hypothetical, but with limited ability to assess heterogeneity in times of COVID-19 between tasks, domains, or person characteristics. We aim to complement the above findings by providing the first (to our knowledge) examination of risk tolerance in times of COVID-19 using a suite of the most commonly used risk-taking measures in behavioral economics and psychology, within a general and a representative sample of the population, and also in relation to real-world risk-taking behavior in times of COVID-19.

## Materials and Methods

### Participants and Procedure

We collected data across two pre-registered (https://osf.io/759df/ and https://osf.io/nk7tb/) online studies during the early 2020 UK lockdown with a total of 1,254 UK residents. In study 1 we collected data from *N* = 955 participants (633 female, M age = 32.6, age range = 18–77). In study 2 we collected data from a representative UK sample of *N* = 299 participants (151 female, M age = 45.6, age range = 18–75), additional demographic information about the participants can be found in Supplementary Material B. Participants for both studies were recruited via *Prolific Academic* to complete the respective Qualtrics-based survey. While the only selection criteria for Study 1 was that participants are UK residents, Study 2 was conducted with a representative sample of UK residents. For their participation, the participants received a fixed payment of GBP 2.00 (Study 1) and GBP 3.00 (Study 2). Moreover, participants had the chance to be paid an additional bonus of up to GBP 81.80 based on two incentive compatible tasks. The average completion times for Studies 1 and 2 were 17.81 min (*SD* = 9.02) and 31.68 min (*SD* = 12.21), respectively. Both studies were pre-registered and conducted in full compliance with the research ethics policy and procedures of the authors' home institution (Ethics Approval number: 07564).

In both studies risk tolerance was elicited using the same four risk tasks. Participants first completed the incentive compatible BART (Lejuez et al., 2002) before completing the self-reported Domain-Specific Risk Taking Task (DOSPERT; Weber et al., 2002). Next, participants completed our second incentive compatible task, which is the BEG multiple lotteries task (Binswanger, 1980; Eckel and Grossman, 2002). Lastly, our participants were presented with the German SOEP self-reported questions for risk-taking (Wagner et al., 2011). Following this, participants completed a short personality inventory and answered a series of demographic and control questions. Additionally, participants in the study with the representative sample (Study 2) answered a range of questions about COVID-19-related risky behaviors selected from the UCL COVID-19 Social Survey (UCL, 2020) and the ICL-YouGov survey on COVID-19 behaviors (ICL, 2020) as measures of real-world risk behavior during the current pandemic.

### Risk Taking Measures

#### Balloon Analog Risk Task (BART)

The BARTis an incentive-compatible computerized measure of risk-taking behavior where participants are presented with a series of balloons and can earn money as they pump up the balloons (Lejuez et al., 2002). For each balloon participants earn a fixed amount per pump. However, as with real balloons, the balloons will burst at some unbeknownst point. In this case the earnings for this particular balloon are lost and the next balloon is presented. Alternatively, participants can choose to stop pumping up the balloon, “bank” the earnings and move on to the next one. While participants are told that the individual balloons will pop at different points, the actual probability distribution is not revealed. Each participant's pay-out for the task depends on the sum of the size of the balloons that did not pop, and risk tolerance is calculated for each participant as the average number of pumps for the balloons that did not pop. This is also known as the Adjusted BART Score. In our study participants are presented with a total of 20 balloons and can earn GBP 0.01 per pump. Their total earnings are calculated after the game and are paid out to the participant with a 1 in 20 chance.

#### Binswanger-Eckel and Grossman Task (BEG)

Another method frequently used to elicit risk tolerance in behavioral and experimental economics is the BEG experimental multiple lotteries task (Binswanger, 1980; Eckel and Grossman, 2002). In this very simple incentivized task, participants choose one of six gambles with risky pay-outs. Each of the six gambles has two potential outcomes that are equally likely to occur (50% chance each). However, the amounts differ between the gambles in two ways: (i) the expected pay-out increases between the gambles and (ii) together with the increasing expected pay-out, the variability of the two outcomes also increases. In addition to the standard choices, we also added a choice to not take part in this gamble at all, as we are aware that some participants might not want to participate in such a gamble at all, e.g., for religious reasons (see Supplementary Material C for our adapted version of the BEG). In our study the lottery pay-out ranged from GBP 28 to GBP 70. The pay-out for each participant was calculated after the game and paid out to the participant with a 1 in 100 chance. Advantages of this task include its very simple nature and the possibility to estimate utility parameter intervals under the assumption of constant relative risk aversion (Charness et al., 2013).

#### Domain-Specific Risk-Taking (DOSPERT)

Some of the most commonly used measures of risk tolerance rely on self-reported willingness to take risk under specific circumstances (Highhouse et al., 2017). For example, the Domain-Specific Risk-Taking (DOSPERT) scale developed by Weber et al. (2002) assesses risk-taking in five specific domains of interest: financial decisions, health/safety, recreational, ethical, and social decisions. Respondents rate the likelihood that they would engage in risky activities such as “skydiving,” “having unprotected sex,” or “not returning a wallet you found that contains $200” on a Likert scale from 1 to 7. Based on their answers an overall score is calculated by participant, as well as a score for each of the five above mentioned domains.

#### German Socioeconomic Panel (SOEP)

Similar to the DOSPERT, the German Socioeconomic Panel (SOEP) measures risk tolerance using a number of self-reported questions on a Likert scale in different domains (Wagner et al., 2011). The SOEP, however, directly asks participants to indicate their “willingness to take risks” in general as well as additionally in five specific contexts (driving a car, financial matters, sport and leisure, career, and health) using a Likert scale from 0 to 10.

### COVID-19 Behavior Measures

In addition to the above risk-taking elicitation methods, participants of study 2 (UK representative sample) also answered a number of questions about their real-world risk-taking behavior using a selection of questions from the UCL COVID-19 Social Survey (UCL, 2020) and the ICL-YouGov survey on COVID-19 behaviors (ICL, 2020). Specifically, we analyzed real-world behaviors such as smoking and drinking as well as a number of directly COVID-19 related behaviors. The COVID-19 behaviors are self-reported behaviors over the previous 7 days (e.g., “*On how many days last week did you leave your house?*” or “*If you left your house last week, on how many of those days did you wear a face mask or covering?*”) and provide a composite precautions index averaging across 20 of such items (see Supplementary Material D for details). In addition, we recorded the number of times they had physical contact outside and inside their household separately, as well as their isolation status. In general, the higher the score for the individual risk behaviors, the more risk is taken, with the exception of the precautions index, and of the frequency of wearing a mask, where a higher score indicates less risk taken (see next section for details).

In the following section, we summarize the results of our analysis. For consistency and comparability of results across models (e.g., forest plots), all regressions have been conducted using linear regression models. In all the estimated linear regression models, we also report the adjusted *R*-squared to allow the reader to assess and compare the goodness-of-fit of each statistical model. We have nonetheless conducted a number of robustness checks in our analyses. First, we have replicated the analyses by including several exogenous control variables in the multiple linear regressions (which generally tend to improve the goodness-of-fit of the empirical estimations). Second, we have also tested each estimated model for possible heteroskedasticity using standard diagnostic tests (e.g., Breusch-Pagan/Cook-Weisberg test for heteroskedasticity) and, in the few cases where the tests led to reject the null hypothesis of homoskedasticity, we have replicated the analyses by running robustness checks using standard appropriate corrections to account for heteroskedasticity (e.g., robust standard errors, weighted linear regression models). Finally, where appropriate, we have also replicated the analyses using a range of appropriate non-linear regression models, such as ordered probit, ordered logit, probit, and logit models. These further sets of robustness checks have substantially confirmed the main findings presented below.

## Results

First, in the data collected from Study 1 and 2 we explored the potential heterogeneity of risk-taking across exogenous characteristics such as gender and age, which have previously been shown to be sources for inter-individual differences in risk-taking. Given the relevance of the individual health status for the risks related to COVID-19, we also looked at the heterogeneity of risk-taking across different levels of self-reported health in both studies.

Second, in Study 2 we looked at heterogeneity in self-reported real-world risk health behaviors at any time (smoking and drinking), and since the start of the COVID-19 pandemic (self-isolation, mask wearing, a composite score of precautionary behaviors). In terms of the real-world risk behaviors in health at any time, smoking, and drinking behavior were recorded as binary variables: being a *Smoker* (1) or not (0); being a *Drinker* (1) or not (0). We selected these variables in the same way they are used and coded in the UCL (2020) COVID-19 survey for the sake of comparability. However, these questions do not allow us for a more differentiated categorization of drinking or smoking behavior (e.g., by number of alcohol units or cigarettes consumed). In terms of the COVID-19 relevant risk behaviors, *Left House* was recorded as a discrete variable that assumes values ranging from 0 to 7 and represents the self-reported number of days on which the participant left the house in the previous week; *Mask Ratio* was recorded as a continuous variable ranging from 0 to 1 calculated as the ratio between the self-reported number of days on which the participant left the house and wore a mask in the previous week, divided by the above defined *Left House*; *Precautions* is a continuous variable on the interval between 1 and 5, which is calculated as the simple average across 20 questions about precautionary COVID-19 behaviors (see Supplementary Material D) where participants self-reported how often they engaged in these behaviors over the past 7 days on a Likert scale from 1 (not at all) to 5 (always); *Contact Outside* referred to the participants' self-reported number of times they have had physical contact with someone outside their household over the previous 7 days, and was recorded as an ordinal variable ranging from 0 to 5 (where: 0 = 0 times, 1 = 1–2 times, 2 = 3–4 times, 3 = 5–9 times, 4 = 10–19 times, and 5 = more than 20 times). *Contact Inside* was calculated in the same way (ordinal variable ranging from 0 to 5) except that participants self-reported the number of times they had physical contact with someone *inside* their own household. Finally, *Isolation Status* was recorded as an ordinal variable ranging from 1 to 5 where participants self-report their isolation status from being in full isolation (1) to leaving the house for a multitude of reasons and not adhering to social distancing in public (5).

Finally, we tested if there was any evidence in support of the risk compensation hypothesis using two data sources. First, by looking at signs of implicit compensation between the self-report health behaviors (at any time and in times of COVID-19) and our elicited risk-taking measures. Second, we looked for explicit signs of compensation in self-reported questions on mask wearing in relation to other COVID-19 risk behaviors. Specifically, participants self-reported if, on days they wore a mask, they engaged less (−1), more (1), or about the same (0) in six behaviors that would put them or others at risk of being exposed to the virus. For our purposes, *Mask Compensation Behavior* was recorded as a continuous variable on the interval between −1 and 1, calculated as the simple arithmetic average across six questions about engaging in COVID-19 risky behavior when wearing a mask (see Supplementary Material E). Thus, a negative *Mask Compensation Behavior* score indicates that a participant is less likely to engage in behaviors that would put them or others at risk of being exposed to the virus on days they wore a mask—which in itself is a risk-reducing behavior.

### Heterogeneity of Risk Taking Across Gender and Age

**Figure 1:**
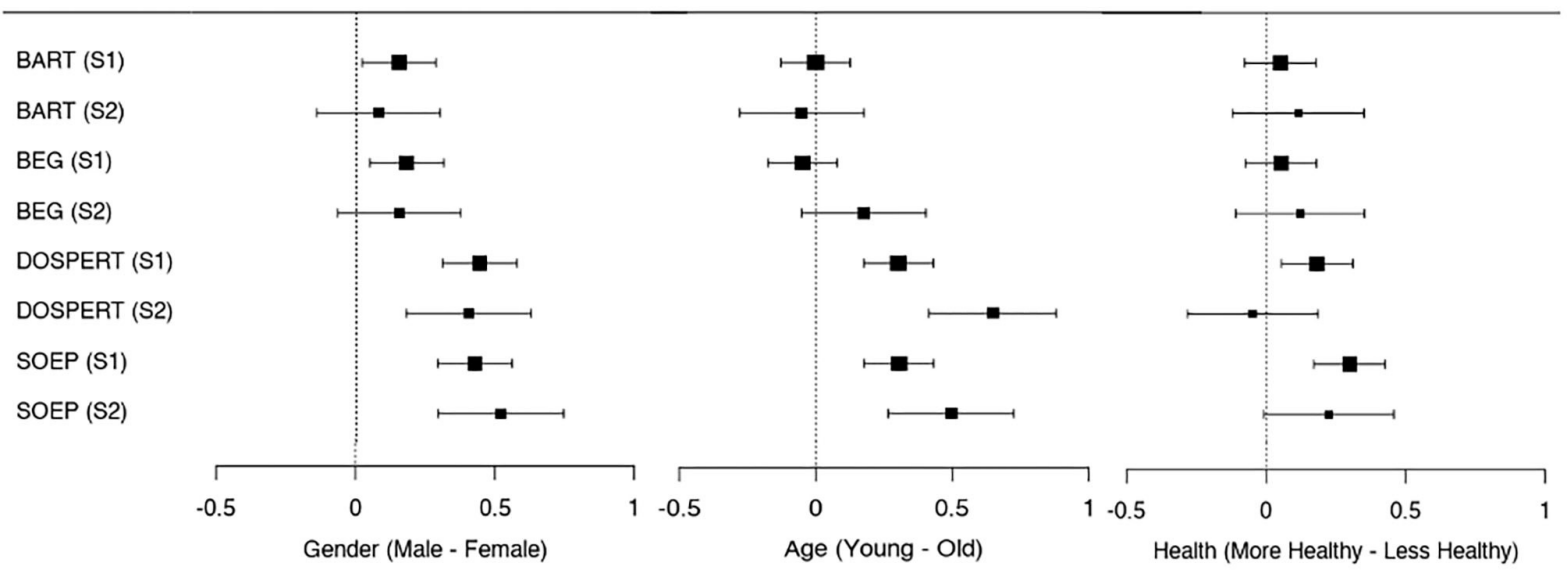
Forest plot for the standardized (z-scored) mean differences with 95% confidence interval across the main risk measures in study 1 (general sample) and study 2 (UK representative sample) by gender, age (classified as young and old by median age), and self-reported health (classified as more and less healthy by median self-reported health). A positive number indicates higher risk tolerance by male participants compared to female participants, by younger compared to older participants, and by healthier compared to less healthy participants, respectively.

Figure 1 summarizes the standardized mean differences for our four main risk measures across Study 1 (S1) and Study 2 (S2) in a series of forest plots. The left-most plot displays differences between male and female participants (a positive number indicates higher risk tolerance by male participants compared to their female counterparts) and shows that male participants displayed a higher degree of risk tolerance than their female counterparts across all four measures and both studies. This finding is statistically significant in all cases except for the two incentive compatible risk-taking tasks (BART and BEG) in Study 2 (UK representative sample; see Supplementary Material F for further detail on domain specific differences).

The middle panel of Figure 1 separates younger and older participants by their respective median age across the two studies (below 30 and above or equal to 30 for Study 1; below 46 and above or equal to 46 for Study 2). In line with the findings from the multiple linear regression model (Table 1), the younger half of the participants displayed significantly higher risk tolerance than the older half of the participants as elicited via the self-reported measures (DOSPERT and SOEP), but no statistically significant differences were found for the incentive-compatible tasks (BART and BEG).

**Table 1:** Linear regression analysis—main risk measures across the two studies regressed on the exogenous participant characteristics.

	**BART (S1)**	**BART (S2)**	**BEG (S1)**	**BEG (S2)**	**DOSPERT (S1)**	**DOSPERT (S2)**	**SOEP (S1)**	**SOEP (S2)**
Intercept	38.150***	34.566***	2.158***	5.163***	4.629***	5.423***	8.603***	9.750***
	(4.457)	(8.570)	(0.536)	(0.993)	(0.245)	(0.446)	(0.761)	(1.347)
Ln (Age)	−1.000	0.088	0.228	−0.530*	−0.506***	−0.708***	−1.164***	−1.366***
	(1.283)	(2.240)	(0.155)	(0.259)	(0.070)	(0.117)	(0.219)	(0.352)
Male	2.030*	1.343	0.303**	0.281	0.355***	0.326***	1.046***	1.211***
	(0.933)	(1.690)	(0.112)	(0.196)	(0.051)	(0.088)	(0.159)	(0.266)
Black	−3.680	−0.233	0.500	−0.008	−0.179	0.051	1.027*	0.810
	(2.728)	(3.380)	(0.332)	(0.390)	(0.150)	(0.176)	(0.466)	(0.532)
Mixed or Multiple	2.750	−5.004	0.395	0.325	−0.269	0.171	−0.504	−0.715
	(2.678)	(4.290)	(0.319)	(0.494)	(0.147)	(0.223)	(0.457)	(0.674)
Asian	−2.230	−1.308	−0.024	−0.746*	−0.151	−0.186	0.133	0.374
	(1.695)	(2.830)	(0.205)	(0.327)	(0.093)	(0.148)	(0.290)	(0.445)
Other ethnic group	−3.200	10.808	−0.393	−1.060	−0.240	0.194	−0.283	−0.110
	(3.889)	(7.380)	(0.484)	(0.850)	(0.214)	(0.384)	(0.664)	(1.160)
Adj. *R*-sq	0.005	−0.005	0.008	0.021	0.092	0.143	0.072	0.110
Obs.	936	291	927	289	936	292	935	292
*F*-Stat	1.720	0.746	2.200	2.020	16.800	9.150	13.100	7.040
*p*-value	0.113	0.613	0.041	0.063	<0.001	<0.001	<0.001	<0.001

Standard error in brackets. ***p < 0.001, **p < 0.01, *p < 0.05.

Next, we looked at gender and age differences in risk tolerance using multiple linear regressions for each risk task and study separately. We explicitly controlled for participants' gender, age, and ethnicity (Table 1). In particular, *ln(Age)* is the natural logarithm of self-reported age; *Male* is a dummy variable that is equal to 1 if the participant self-reported to be male, and 0 otherwise; *Asian, Black, Mixed, or Multiple*, and *Other Ethnic Group* are dummy variables equal to 1 if the participant self-reported to be part of the respective ethnic group, and equal to 0 otherwise—with self-reported to be *White* as the reference group.

We observed gender effects for all risk-taking tasks: male participants displayed a higher degree of risk tolerance than female participants across all four measures in both studies. We also found that risk-tolerance elicited via our self-reported measures (DOSPERT and SOEP) decreased significantly with age in both our studies, while there were no age-related significant differences in risk-taking in the incentive-compatible tasks (BART and BEG). We did not observe any clear pattern for differences between ethnic groups.

### Heterogeneity of Risk Taking Across Self-Reported Health

The most right panel of Figure 1 (Health) summarizes the standardized mean differences for our four main risk-taking measures with respect to self-reported health. We separated healthier and less healthy participants by their self-reported health median score (above or equal to 81 and below 81 for Study 1; above or equal to 80 and below 80 for Study 2). The differences depicted are between healthier and less healthy participants where a positive number indicates higher risk tolerance by healthier participants compared to their less healthy counterparts. As can be seen, only in the self-reported measures in Study 1 (DOSPERT and SOEP) risk-taking is significantly higher in healthier participants.

### Correlation Between Risk Measures

Additionally, we display the overall correlation scores for our risk measures for Study 1 and Study 2 in Tables 2, 3, respectively. While we do not find a clear correlation pattern of the BART with the other risk measures, we find a weak positive correlation between the BEG and the DOSPERT as well as the SOEP, and a moderate positive correlation between the DOSPERT and the SOEP.

**Table 2:** Correlation analysis—Pearson correlation coefficients for the main risk measures for study 1.

**Study 1**	**BART**	**BEG**	**DOSPERT**	**SOEP**
BART	—			
BEG	0.067*	—		
DOSPERT	0.026	0.140***	—	
SOEP	0.026	0.215***	0.482***	—

***p < 0.001, **p < 0.01, *p < 0.05.

**Table 3:** Correlation analysis—Pearson correlation coefficients for the main risk measures for study 2.

**Study 2**	**BART**	**BEG**	**DOSPERT**	**SOEP**
BART	—			
BEG	0.170**	—		
DOSPERT	0.151**	0.203***	—	
SOEP	−0.004	0.229***	0.544***	—

***p < 0.001, **p < 0.01, *p < 0.05.

### Heterogeneity of Real-World Health and COVID-19 Risky Behaviors

**Figure 2:**
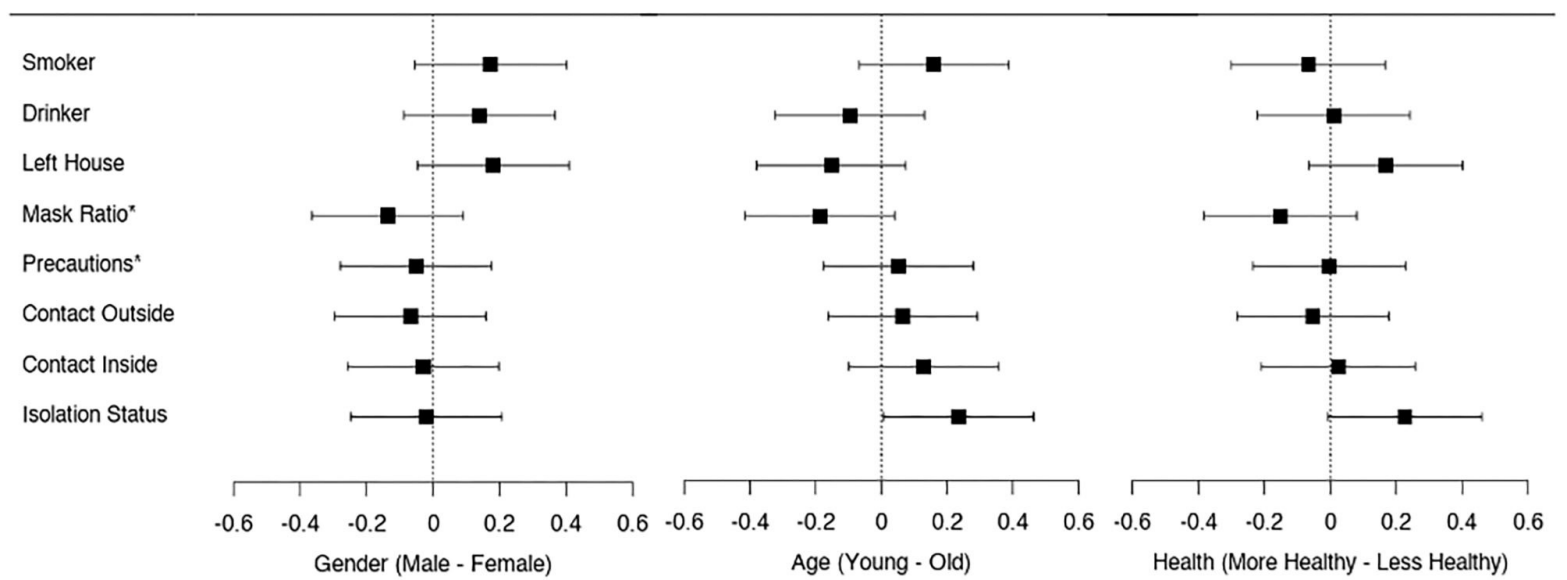
Forest plot for the standardized (z-scored) mean differences with 95% confidence interval across the self-reported risky behaviors in study 2 (UK representative sample) by gender, age (classified as young and old by median age), and self-reported health (classified as more and less healthy by median self-reported health). A positive number indicates higher real-world risk taking by male participants compared to female participants, by younger compared to older participants, and by healthier compared to less healthy participants, respectively. *Mask Ratio and Precautions have been inverted by multiplying the corresponding group means by minus one so that a higher score indicates higher risk taking.

In contrast to the findings for the risk-taking measures, we did not find any significant evidence for gender differences in the self-reported risk-taking behaviors in our representative sample (see Figure 2 and Table 4). While the impact of age is generally in line with the risk-taking measures,—i.e., risk-taking decreases with age—this result is only statistically significant for the isolation status. We did find some evidence for differences based on ethnicity among our participants. More specifically, we found evidence that Black and Asian participants are less risk taking on some of the measured behaviors compared to their white counterparts. This may be related to the fact that Black and other ethnic minorities have higher baseline risks of getting infected by the COVID-19 because they are, for example, more likely to work in frontline jobs, or to live in multi-generational households (Bowyer et al., 2020; Mathur et al., 2020).

**Table 4:** Linear regression analysis—self-reported real-world risky behaviors for study 2 (UK representative sample) regressed on the exogenous participant characteristics.

	**Smoker**	**Drinker**	**Left house**	**Mask ratio**	**Precautions**	**Contact outside**	**Contact inside**	**Isolation status**
Intercept	0.310	0.823**	2.597*	0.505	3.174***	1.405*	4.316***	4.318***
	(0.205)	(0.266)	(1.312)	(0.266)	(0.473)	(0.678)	(1.167)	(0.595)
ln(Age)	−0.053	−0.026	0.333	−0.058	0.091	−0.132	−0.283	−0.393*
	(0.054)	(0.069)	(0.343)	(0.070)	(0.124)	(0.177)	(0.305)	(0.155)
Male	0.062	0.070	0.416	0.045	0.022	−0.074	−0.040	−0.024
	(0.040)	(0.052)	(0.259)	(0.053)	(0.093)	(0.134)	(0.231)	(0.117)
Black	−0.048	−0.262*	−1.226*	0.394***	0.210	0.216	−1.308**	−0.215
	(0.081)	(0.105)	(0.517)	(0.107)	(0.187)	(0.267)	(0.460)	(0.235)
Mixed or Multiple	0.020	−0.094	−0.036	0.038	0.261	0.203	−0.416	−0.019
	(0.102)	(0.133)	(0.656)	(0.129)	(0.237)	(0.339)	(0.583)	(0.298)
Asian	−0.046	−0.263**	−1.146**	0.338***	0.507**	−0.123	0.298	−0.146
	(0.069)	(0.088)	(0.433)	(0.090)	(0.156)	(0.224)	(0.385)	(0.196)
Other ethnic group	0.082	−0.272	−2.649*	0.646	0.152	−0.185	0.634	−1.514**
	(0.176)	(0.229)	(1.128)	(0.438)	(0.408)	(0.583)	(1.003)	(0.512)
Adj. *R*-sq	−0.006	0.035	0.051	0.083	0.020	−0.013	0.017	0.026
Obs.	291	292	290	273	292	289	290	292
*F*-Stat	0.731	2.750	3.570	5.130	1.980	0.391	1.840	2.290
*p*-value	0.625	0.013	0.002	<0.001	0.068	0.885	0.091	0.036

Standard error in brackets. ***p < 0.001, **p < 0.01, *p < 0.05.

### Correlation Between Real-World Health and COVID-19 Risky Behaviors

As can be seen in Table 5, being a Drinker or Smoker has virtually no correlation to the COVID-19 risky behaviors. However, we find that out of a total of 15 pairwise correlations between the COVID-19 risky behaviors, 11 correlations are positive and statistically significant (i.e., more risk-taking in one COVID-19 behavior is associated with more risk-taking in another COVID-19 behavior), while none is significantly negative.

**Table 5:** Correlation analysis—Pearson correlation coefficients for self-reported real-world risky behaviors for study 2 (UK representative sample).

	**Drinker**	**Smoker**	**Left house**	**Mask ratio**	**Precautions**	**Contact outside**	**Contact inside**	**Isolation status**
Drinker	—							
Smoker	0.087	—						
Left House	0.081	0.042	—					
Mask Ratio	−0.044	0.022	−0.358***	—				
Precautions	−0.061	−0.018	−0.27***	0.346***	—			
Contact Outside	−0.057	−0.013	0.227***	0.047	−0.127*	—		
Contact Inside	0.059	−0.139*	0.069	−0.149*	−0.139*	0.090	—	
Isolation Status	0.028	0.028	0.391***	−0.215***	−0.405***	0.268***	0.093	—

***p < 0.001, **p < 0.01, *p < 0.05.

### Risk-Taking Measures as Predictors of Health and COVID-19 Risky Behaviors

Real-world risk-taking behavior is difficult to measure and assess, and risk tolerance elicited in a lab-setting is often used as a predictor for real-world risky behavior (Sutter et al., 2013; Galizzi et al., 2016a; Charness et al., 2020). Having elicited risk-tolerance using our four risk-taking measures and having collected self-reported COVID-19 real-world risk-taking behavior in Study 2 (UK representative sample), we next investigated the predictive power of the risk-taking measures in the context of COVID-19 risk-taking behavior (see Tables 6, 7).

**Table 6:** Linear regression analysis—self-reported real-world risky behaviors for study 2 (UK representative sample) regressed on the individual risk measures.

	**Smoker**	**Drinker**	**Left house**	**Mask ratio**	**Precautions**	**Contact outside**	**Contact inside**	**Isolation status**
Intercept	0.128*	0.581***	3.850***	0.306***	3.810***	0.864***	3.028***	2.723***
	(0.053)	(0.070)	(0.350)	(0.073)	(0.121)	(0.174)	(0.306)	(0.155)
BART	0.000	0.003	−0.001	0.002	−0.005	0.001	0.004	0.002
	(0.001)	(0.002)	(0.009)	(0.002)	(0.003)	(0.005)	(0.008)	(0.004)
Adj. *R*-sq	−0.003	0.007	−0.003	0.000	0.006	−0.003	−0.003	−0.003
Obs.	296	297	294	277	296	293	294	297
*F*-Stat	0.016	3.220	0.004	1.090	2.790	0.016	0.240	0.166
*p*-value	0.899	0.074	0.952	0.297	0.096	0.899	0.624	0.684
Intercept	0.101*	0.670***	3.303***	0.417***	3.835***	0.771***	3.086***	2.428***
	(0.042)	(0.057)	(0.278)	(0.059)	(0.099)	(0.139)	(0.246)	(0.123)
BEG	0.010	0.008	0.162*	−0.013	−0.069*	0.035	0.022	0.110**
	(0.012)	(0.016)	(0.077)	(0.016)	(0.027)	(0.038)	(0.068)	(0.034)
Adj. *R*-sq	−0.001	−0.002	0.012	−0.001	0.018	−0.001	−0.003	0.031
Obs.	297	298	295	278	297	294	295	298
*F*-Stat	0.794	0.263	4.470	0.657	6.500	0.816	0.103	10.500
*p*-value	0.374	0.609	0.035	0.418	0.011	0.367	0.748	0.001
Intercept	−0.077	0.420***	3.266***	0.303**	4.106***	0.577*	2.897***	2.397***
	(0.073)	(0.098)	(0.495)	(0.103)	(0.173)	(0.246)	(0.436)	(0.217)
DOSPERT	0.072**	0.095**	0.190	0.024	−0.169**	0.104	0.089	0.131
	(0.024)	(0.032)	(0.164)	(0.034)	(0.057)	(0.081)	(0.144)	(0.072)
Adj. *R*-sq	0.027	0.025	0.001	−0.002	0.025	0.002	−0.002	0.008
Obs.	297	298	295	278	297	294	295	298
*F*-Stat	9.070	8.530	1.350	0.520	8.750	1.640	0.380	3.360
*p*-value	0.003	0.004	0.246	0.471	0.003	0.201	0.538	0.068
Intercept	0.056	0.569***	3.290***	0.264***	3.694***	0.746***	3.235***	2.471***
	(0.048)	(0.064)	(0.321)	(0.067)	(0.113)	(0.160)	(0.283)	(0.140)
SOEP	0.015	0.024*	0.100	0.021	−0.015	0.026	−0.015	0.058*
	(0.008)	(0.011)	(0.055)	(0.012)	(0.020)	(0.028)	(0.049)	(0.024)
Adj. *R*-sq	0.008	0.012	0.008	0.008	−0.001	0.000	−0.003	0.016
Obs.	297	298	295	278	297	294	295	298
*F*-Stat	3.280	4.720	3.290	3.260	0.602	0.854	0.096	5.800
*p*-value	0.071	0.031	0.071	0.072	0.438	0.356	0.757	0.017
Intercept	−0.073	0.346**	3.054***	0.248*	4.270***	0.546	2.891***	2.207***
	(0.085)	(0.114)	(0.567)	(0.121)	(0.194)	(0.286)	(0.502)	(0.249)
BART	−0.001	0.003	−0.001	0.002	−0.004	0.000	0.004	0.000
	(0.001)	(0.002)	(0.009)	(0.002)	(0.003)	(0.005)	(0.008)	(0.004)
BEG	0.003	−0.001	0.171*	−0.020	−0.059*	0.016	0.006	0.100**
	(0.012)	(0.017)	(0.081)	(0.017)	(0.028)	(0.041)	(0.072)	(0.036)
DOSPERT	0.071*	0.069	−0.047	−0.008	−0.161*	0.086	0.095	0.017
	(0.030)	(0.040)	(0.198)	(0.041)	(0.068)	(0.100)	(0.175)	(0.087)
SOEP	0.002	0.011	0.074	0.026	0.028	0.010	−0.029	0.040
	(0.010)	(0.013)	(0.066)	(0.014)	(0.023)	(0.033)	(0.059)	(0.029)
Adj. *R*-sq	0.018	0.022	0.011	0.006	0.037	−0.007	−0.011	0.033
Obs.	293	294	291	274	293	290	291	294
*F*-Stat	2.340	2.670	1.830	1.420	3.840	0.503	0.195	3.510
*p*-value	0.055	0.032	0.124	0.228	0.005	0.733	0.941	0.008

Standard error in brackets. ***p < 0.001, **p < 0.01, *p < 0.05.

**Table 7:** Correlation analysis—Pearson correlation coefficients for self-reported real-world risky behaviors for study 2 (UK representative sample) and the individual risk measures.

	**Drinker**	**Smoker**	**Left house**	**Mask ratio**	**Precautions**	**Contact outside**	**Contact inside**	**Isolation status**
BART	0.104	0.007	−0.004	0.045	−0.087	0.007	0.029	0.024
BEG	0.043	0.045	0.136*	−0.054	−0.109	0.038	0.005	0.187**
DOSPERT	0.167**	0.172**	0.068	0.057	−0.149**	0.075	0.036	0.106
SOEP	0.125*	0.105	0.105	0.120*	0.003	0.054	−0.018	0.138*

***p < 0.001, **p < 0.01, *p < 0.05.

We find that the BART measure for risk taking has virtually no predictive power of the real-world risk-taking behaviors in our study. This result remains robust when controlling for demographic characteristics and for multiple measures of risk taking at the same time (see Supplementary Material G, Tables G1, G5).

The BEG, on the other hand, appears to have predictive power over only one of our risk-related behaviors, namely the isolation status: respondents with a higher BEG score are significantly less likely to isolate and stay at home. The significant effect is robust when controlling for the respondents' exogenous characteristics (gender, age, ethnicity) and for multiple measures of risk taking at the same time (see Supplementary Material G, Tables G2, G5).

Also the SOEP is positively associated with the isolation status but the effect is only marginally statistically significant and is not robust across various specifications (e.g., controlling for multiple measures of risk taking at the same time; see Supplementary Material G, Tables G4, G5). Similarly, participants taking more risks in the BEG task score lower on our *Precautions* index, but the effect is only marginally statistically significant.

The DOSPERT has predictive power over the precautionary index: respondents with a higher DOSPERT score have a significantly lower *Precautions* index. The significant effect is robust across further specifications controlling for the respondents' exogenous characteristics (gender, age, ethnicity) and for multiple measures of risk taking at the same time see Supplementary Material G, Tables G3, G5).

As we are investigating the predictive power of the risk-taking measures in the context of health-related real-world behaviors, we also analyzed the predictive power of the relevant health scales (see Supplementary Material H). We find—as expected—that the health scales for the DOSPERT and the SOEP improve the predictive power for health-related real-world behaviors compared to the respective overall or general score, this is particularly the case for the SOEP.

### Self-Report Risk Compensation Between Mask Wearing and COVID-19 Risky Behavior

We finally investigate if there is evidence in support of the risk compensation hypothesis among our participants. As discussed, in order to test this hypothesis, we focus on the first behavioral channel explained above and we specifically inspect compensation between mask wearing and COVID-19 precaution behaviors. Recent discussions of compensation surrounding this particular behavior are contrasting (Greenhalgh et al., 2020). For example, a recent online study reported higher risk taking when wearing a mask, measured in terms of reduced social distancing in a hypothetical online task (Luckman et al., 2020), whilst a recent rapid review of the COVID-19 relevant risk compensation literature (with a particular focus on the mask compensation hypothesis) concludes that the hypothesis is a “*dead horse that no longer needs to be beaten*” and that it needs “*burying to try to prevent the continued threat it poses slowing the adoption of effective public health interventions*” (Mantzari et al., 2020, p. 3).

For this analysis, we used the data collected in Study 2 (UK representative sample) and looked at our *Mask Compensation Behavior* index, which is the average of self-reported scores to six questions about COVID-19 related behavior *when* wearing a mask (see Materials and Methods section for detail; see Supplementary Material E for the questions). More specifically, we performed a two-tailed *t*-test to identify whether participants were more or less likely to engage in COVID-19 risky behaviors when wearing a mask.

Overall, we found that participants are, on average, significantly *less* likely to engage in COVID-19-related risky behaviors when wearing a mask as measured by our *Mask Compensation Behavior* index [*M* = −0.171, *SD* =0.320, *t*_(251)_ = −8.488, *p* < 0.001]. Reviewing each of the six items independently did also not provide any evidence for risk compensation. This is consistent with the recent experimental findings by Seres et al. (2020) and, in sum, is exactly the opposite of what would be postulated by the risk homeostasis hypothesis.

When controlling for participant characteristics, we found that the overall finding is driven by Asian and Black participants. We do not find any gender- or age-related differences in the propensity to engage in risky behaviors when wearing a mask (see Supplementary Material I).

## Conclusions

We conducted two pre-registered online studies during the COVID-19 pandemic and the early 2020 lockdown with a total of 1,254 UK residents (one of which with a UK representative sample) to elicit risk-tolerance using four of the most widely applied risk-taking tasks in behavioral economics and psychology. Specifically, participants completed the incentive-compatible BART and BEG tasks, as well as the DOSPERT and the SOEP self-reported questions for risk-taking. In addition, participants in the UK representative sample answered a range of questions about COVID-19-related risky behaviors selected from the UCL COVID-19 Social Survey and the ICL-YouGov survey on COVID-19 behaviors.

We report four sets of main findings. First, consistently with findings in pre-COVID-19 times (Croson and Gneezy, 2009; Dohmen et al., 2011, 2017; Rolison et al., 2012; Falk et al., 2018; König-Kersting and Trautmann, 2018), we found that risk tolerance elicited via our four risk measures during the UK lockdown was (i) higher in men than in women and (ii) decreased with age. Notebly, we did not find any significant evidence for gender or age effects in the self-reported everyday health or COVID-19 specific risky behaviors in our representative sample.

Second, undocumented for pre-COVID-19 times, we found that healthier participants in Study 1 displayed significantly higher risk tolerance in self-reported risk-taking measures. One interpretation is that the high background uncertainty due to COVID-19 could lead to diminished sensitivity to small foreground risks, which thus become less salient (Galizzi et al., 2020; Harrison et al., 2020). This, in turn, could lead the healthier segments of the population—for example the respondents with no pre-existing health conditions—to engage more with risk-taking in some of our tasks. Further research is needed to replicate the test of this intriguing possibility.

Third, overall, we found no systematic nor robust patterns of association between the COVID-19-related risky behaviors and the four risk-taking tasks in our samples. Two notable exceptions are that respondents with a higher BEG score are significantly less likely to isolate and stay at home; and that respondents with a higher DOSPERT score have a significantly lower precautionary index. None of the other associations are statistically significant or robust. For smoking and drinking, we found that only the DOSPERT has a significant and robust association with these two real-world risk behaviors. Most notably, we did not find a significant association between smoking and the BART. While our results might be difficult to interpret as *drinker* and *smoker* are binary variables, the BART has previously been found to predict smoking behavior measured as a binary variable (Lejuez et al., 2003). Overall, therefore, our (lack of) evidence is in line with the pessimistic view laid out by Trautmann (2016) and Friedman et al. (2017) on the weak empirical links between risk-taking measures and real behavior.

Finally, we found no evidence in support of the so-called “risk compensation” hypothesis. We empirically tested such a hypothesis through two distinct behavioral channels behind it and found no evidence in either of them. If anything, it appears that participants who took greater risk in regard to their everyday health (drinking, smoking) or in relation to being exposed to COVID-19 (e.g., lower levels of isolation, mask wearing, or taking precautions) also exhibited higher risk-tolerance in our experimental and self-reported risk-taking measures. This is consistent with recent reviews conducted at the start of COVID-19 pandemic (Mantzari et al., 2020), with direct experimental evidence from the field (Seres et al., 2020), as well as with results from pre-COVID-19 times (Cowling et al., 2009; Aiello et al., 2012; Kasting et al., 2016; Madhivanan et al., 2016; Pless, 2016) which all find no significant evidence in support of the risk compensation hypothesis. Further research could look at the relationships between the various specific COVID-19-related risk behaviors. For this purpose, the correlation matrix displaying relationships between the different COVID-19-relevant risky behaviors (Table 5) can serve as a starting point. As can be seen, out of a total of 15 pairwise correlations, 11 correlations are positive and statistically significant (i.e., more risk-taking in one COVID-19 behavior is associated with more risk-taking in another COVID-19 behavior), while none is significantly negative. We see this as further suggestive evidence against the risk compensation hypothesis in our sample.

Other additional research is required to explore the role of heterogeneity in other individual and household characteristics (e.g., wealth, income, education, employment status) and to further investigate inter-individual and domain specific differences in risk taking during the unprecedented time of the global COVID-19 pandemic. As a potential starting point on the latter point, Supplementary Material F reports an analysis of risk taking in the DOSPERT and SOEP tasks separated between risk domains (e.g., health, social, financial, recreational). Moreover, the scope is evident for designing and validating new measures of risk-taking that more closely and effectively reflect and predict COVID-19-related real-world behaviors, in order to inform policy and help the design of targeted behavioral interventions.

## Data Availability Statement

The raw data supporting the conclusions of this article will be made available by the authors, without undue reservation upon request.

## Ethics Statement

Ethical review and approval was not required for the study on human participants in accordance with the local legislation and institutional requirements. The patients/participants provided their written informed consent to participate in this study.

## Author Contributions

BG led the survey design, data analysis and writing. MG led the data collection. MG and JS both contributed to data analysis and writing. All authors contributed to the article, conceptualization, methodology, survey design, funding of the study, and approved the submitted version.

## Conflict of Interest

BG was employed by the company Salient Behavioural Consultants Ltd. The remaining authors declare that the research was conducted in the absence of any commercial or financial relationships that could be construed as a potential conflict of interest.
