# Supplementary material for: Heterogeneity in Risk-Taking During the COVID-19 Pandemic: Evidence From the UK Lockdown
Source: Front Psychol. 2021 Apr 1;12:643653. doi: 10.3389/fpsyg.2021.643653 (PMC8046913; doi:10.3389/fpsyg.2021.643653)
Supplement: Supplementary file 1 [file Table_1.DOCX]

**Supplementary Materials A: Risk compensation between risk tasks and real-world risk behaviours**

For the sake of completeness and transparency, here we also report the empirical results related to testing the risk compensation hypothesis via the second possible channel described in the manuscript, namely the empirical test of the conjecture that respondents who take less health or Covid-19-related risks in the real world (e.g. by wearing a mask, self-isolating) take *more* risks in the risk-taking tasks in our online experiment.

We test this by analysing the relationship between the everyday real-world health (smoking and drinking) and Covid-19 risky behaviours (e.g. self-isolation, precautions, mask wearing) and our elicited risk-taking measures. In each model, we regressed the respective risk-taking measure on each of the eight risky behaviours individually (Model 1- Model 8), followed by a model where the risk-taking measure is regressed on all eight risky behaviours collectively (Model 9). We present this for the BART (Table A1), BEG (Table A2), DOSPERT (Table A3) and SOEP (Table A4), respectively.

Table A1: Linear regression analysis – BART for study 2 (UK representative sample) regressed on the self-reported real-world risky behaviours. Standard error in brackets. *** p < 0.001, ** p < 0.01, * p < 0.05.

|  |  |  |  |  |  |  |  |  |  |
| --- | --- | --- | --- | --- | --- | --- | --- | --- | --- |
| **BART** | **Model 1** | **Model 2** | **Model 3** | **Model 4** | **Model 5** | **Model 6** | **Model 7** | **Model 8** | **Model 9** |
|  |  |  |  |  |  |  |  |  |  |
|  |  |  |  |  |  |  |  |  |  |
| Intercept | 35.320*** | 33.050*** | 35.454*** | 34.760*** | 41.660*** | 35.239*** | 34.623*** | 34.391*** | 42.303*** |
|  | (0.904) | (1.520) | (1.659) | (1.120) | (3.910) | (1.076) | (1.589) | (2.455) | (7.279) |
| Smoker | 0.314 |  |  |  |  |  |  |  | 0.930 |
|  | (2.465) |  |  |  |  |  |  |  | (2.592) |
| Drinker |  | 3.260 |  |  |  |  |  |  | 2.515 |
|  |  | (1.820) |  |  |  |  |  |  | (1.938) |
| Left House |  |  | -0.023 |  |  |  |  |  | -0.139 |
|  |  |  | (0.373) |  |  |  |  |  | (0.462) |
| Mask Ratio |  |  |  | 1.980 |  |  |  |  | 3.611 |
|  |  |  |  | (1.900) |  |  |  |  | (2.216) |
| Precautions |  |  |  |  | -1.760 |  |  |  | -2.411 |
|  |  |  |  |  | (1.050) |  |  |  | (1.360) |
| Contact Outside |  |  |  |  |  | 0.096 |  |  | -0.253 |
|  |  |  |  |  |  | (0.752) |  |  | (0.808) |
| Contact Inside |  |  |  |  |  |  | 0.209 |  | 0.182 |
|  |  |  |  |  |  |  | (0.426) |  | (0.459) |
| Isolation Status |  |  |  |  |  |  |  | 0.338 | -0.397 |
|  |  |  |  |  |  |  |  | (0.829) | (1.026) |
| Adj. R-sq | -0.003 | 0.007 | -0.003 | 0.000 | 0.006 | -0.003 | -0.003 | -0.003 | -0.004 |
| Obs. | 296 | 297 | 294 | 277 | 296 | 293 | 294 | 297 | 275 |
| F-Stat | 0.016 | 3.220 | 0.004 | 1.090 | 2.790 | 0.016 | 0.240 | 0.166 | 0.868 |
| p-value | 0.899 | 0.074 | 0.952 | 0.297 | 0.096 | 0.899 | 0.624 | 0.684 | 0.544 |

Table A2: Linear regression analysis – BEG for study 2 (UK representative sample) regressed on the self-reported real-world risky behaviours. Standard error in brackets. *** p < 0.001, ** p < 0.01, * p < 0.05.

|  |  |  |  |  |  |  |  |  |  |
| --- | --- | --- | --- | --- | --- | --- | --- | --- | --- |
| **BEG** | **Model 1** | **Model 2** | **Model 3** | **Model 4** | **Model 5** | **Model 6** | **Model 7** | **Model 8** | **Model 9** |
|  |  |  |  |  |  |  |  |  |  |
|  |  |  |  |  |  |  |  |  |  |
| Intercept | 3.204*** | 3.121*** | 2.838*** | 3.357*** | 4.446*** | 3.178*** | 3.220*** | 2.367*** | 3.914*** |
|  | (0.105) | (0.176) | (0.192) | (0.132) | (0.449) | (0.126) | (0.187) | (0.281) | (0.815) |
| Smoker | 0.221 |  |  |  |  |  |  |  | 0.156 |
|  | (0.286) |  |  |  |  |  |  |  | (0.303) |
| Drinker |  | 0.157 |  |  |  |  |  |  | 0.072 |
|  |  | (0.212) |  |  |  |  |  |  | (0.226) |
| Left House |  |  | 0.102* |  |  |  |  |  | 0.021 |
|  |  |  | (0.043) |  |  |  |  |  | (0.054) |
| Mask Ratio |  |  |  | -0.152 |  |  |  |  | 0.171 |
|  |  |  |  | (0.225) |  |  |  |  | (0.260) |
| Precautions |  |  |  |  | -0.337** |  |  |  | -0.342* |
|  |  |  |  |  | (0.121) |  |  |  | (0.156) |
| Contact Outside |  |  |  |  |  | 0.058 |  |  | -0.065 |
|  |  |  |  |  |  | (0.088) |  |  | (0.095) |
| Contact Inside |  |  |  |  |  |  | 0.004 |  | -0.010 |
|  |  |  |  |  |  |  | (0.050) |  | (0.054) |
| Isolation Status |  |  |  |  |  |  |  | 0.310** | 0.177 |
|  |  |  |  |  |  |  |  | (0.095) | (0.120) |
| Adj. R-sq | -0.001 | -0.002 | 0.015 | -0.002 | 0.022 | -0.002 | -0.003 | 0.032 | 0.017 |
| Obs. | 294 | 295 | 292 | 275 | 294 | 291 | 292 | 295 | 273 |
| F-Stat | 0.596 | 0.550 | 5.490 | 0.456 | 7.720 | 0.429 | 0.006 | 10.600 | 1.600 |
| p-value | 0.441 | 0.459 | 0.020 | 0.500 | 0.006 | 0.513 | 0.939 | 0.001 | 0.125 |

Table A3: Linear regression analysis – DOSPERT for study 2 (UK representative sample) regressed on the self-reported real-world risky behaviours. Standard error in brackets. *** p < 0.001, ** p < 0.01, * p < 0.05.

|  |  |  |  |  |  |  |  |  |  |
| --- | --- | --- | --- | --- | --- | --- | --- | --- | --- |
| **DOSPERT** | **Model 1** | **Model 2** | **Model 3** | **Model 4** | **Model 5** | **Model 6** | **Model 7** | **Model 8** | **Model 9** |
|  |  |  |  |  |  |  |  |  |  |
|  |  |  |  |  |  |  |  |  |  |
| Intercept | 2.859*** | 2.706*** | 2.827*** | 2.918*** | 3.531*** | 2.874*** | 2.877*** | 2.675*** | 3.378*** |
|  | (0.050) | (0.084) | (0.092) | (0.063) | (0.213) | (0.060) | (0.088) | (0.137) | (0.375) |
| Smoker | 0.411** |  |  |  |  |  |  |  | 0.428** |
|  | (0.137) |  |  |  |  |  |  |  | (0.140) |
| Drinker |  | 0.295** |  |  |  |  |  |  | 0.235* |
|  |  | (0.101) |  |  |  |  |  |  | (0.104) |
| Left House |  |  | 0.024 |  |  |  |  |  | -0.007 |
|  |  |  | (0.021) |  |  |  |  |  | (0.025) |
| Mask Ratio |  |  |  | 0.077 |  |  |  |  | 0.213 |
|  |  |  |  | (0.107) |  |  |  |  | (0.120) |
| Precautions |  |  |  |  | -0.170** |  |  |  | -0.208** |
|  |  |  |  |  | (0.058) |  |  |  | (0.071) |
| Contact Outside |  |  |  |  |  | 0.054 |  |  | 0.016 |
|  |  |  |  |  |  | (0.042) |  |  | (0.044) |
| Contact Inside |  |  |  |  |  |  | 0.015 |  | 0.014 |
|  |  |  |  |  |  |  | (0.024) |  | (0.025) |
| Isolation Status |  |  |  |  |  |  |  | 0.085 | -0.004 |
|  |  |  |  |  |  |  |  | (0.046) | (0.055) |
| Adj. R-sq | 0.027 | 0.025 | 0.001 | -0.002 | 0.025 | 0.002 | -0.002 | 0.008 | 0.068 |
| Obs. | 297 | 298 | 295 | 278 | 297 | 294 | 295 | 298 | 276 |
| F-Stat | 9.070 | 8.530 | 1.350 | 0.520 | 8.750 | 1.640 | 0.380 | 3.360 | 3.510 |
| p-value | 0.003 | 0.004 | 0.246 | 0.471 | 0.003 | 0.201 | 0.538 | 0.068 | < .001 |

Table A4: Linear regression analysis – SOEP for study 2 (UK representative sample) regressed on the self-reported real-world risky behaviours. Standard error in brackets. *** p < 0.001, ** p < 0.01, * p < 0.05.

|  |  |  |  |  |  |  |  |  |  |
| --- | --- | --- | --- | --- | --- | --- | --- | --- | --- |
| **SOEP** | **Model 1** | **Model 2** | **Model 3** | **Model 4** | **Model 5** | **Model 6** | **Model 7** | **Model 8** | **Model 9** |
|  |  |  |  |  |  |  |  |  |  |
|  |  |  |  |  |  |  |  |  |  |
| Intercept | 5.190*** | 4.824*** | 4.875*** | 5.166*** | 5.772*** | 5.201*** | 5.379*** | 4.365*** | 4.362*** |
|  | (0.149) | (0.250) | (0.271) | (0.183) | (0.639) | (0.176) | (0.261) | (0.403) | (1.122) |
| Smoker | 0.735 |  |  |  |  |  |  |  | 0.695 |
|  | (0.406) |  |  |  |  |  |  |  | (0.420) |
| Drinker |  | 0.652* |  |  |  |  |  |  | 0.453 |
|  |  | (0.300) |  |  |  |  |  |  | (0.312) |
| Left House |  |  | 0.111 |  |  |  |  |  | 0.085 |
|  |  |  | (0.061) |  |  |  |  |  | (0.074) |
| Mask Ratio |  |  |  | 0.562 |  |  |  |  | 0.835* |
|  |  |  |  | (0.311) |  |  |  |  | (0.359) |
| Precautions |  |  |  |  | -0.134 |  |  |  | -0.142 |
|  |  |  |  |  | (0.173) |  |  |  | (0.213) |
| Contact Outside |  |  |  |  |  | 0.114 |  |  | -0.035 |
|  |  |  |  |  |  | (0.124) |  |  | (0.131) |
| Contact Inside |  |  |  |  |  |  | -0.022 |  | -0.051 |
|  |  |  |  |  |  |  | (0.070) |  | (0.074) |
| Isolation Status |  |  |  |  |  |  |  | 0.328* | 0.234 |
|  |  |  |  |  |  |  |  | (0.136) | (0.165) |
| Adj. R-sq | 0.008 | 0.012 | 0.008 | 0.008 | -0.001 | 0.000 | -0.003 | 0.016 | 0.028 |
| Obs. | 297 | 298 | 295 | 278 | 297 | 294 | 295 | 298 | 276 |
| F-Stat | 3.280 | 4.720 | 3.290 | 3.260 | 0.602 | 0.854 | 0.096 | 5.800 | 2.000 |
| p-value | 0.071 | 0.031 | 0.071 | 0.072 | 0.438 | 0.356 | 0.757 | 0.017 | 0.047 |

We did not observe any evidence for risk compensation or risk homeostasis in our data. In fact we observed some trends in the opposite direction: we found that being less likely to self-isolate (a highly risky behaviour for being exposed to Covid-19) is significantly and positively associated with higher BEG scores, with the effect being robust with the introduction of control variables (Table A6). Similarly, being classed a *Drinker* (self-reported consumption of one or more drinks) is significantly positively associated with high scores on the DOSPERT, and adopting precautions is significantly negatively associated with the DOSPERT score. Both effects are robust to the introduction of control variables (see Table A7).

So, while the evidence is mixed, on balance we found that participants who reported taking greater risks in real-life health or Covid-19 behaviours also tended to exhibit higher - not lower - risk-tolerance in our online risk-taking tasks. We therefore find no evidence in support of the risk compensation hypothesis in our data even when this hypothesis is empirically tested in terms of the second channel described above.

Table A5: Linear regression analysis – BART for study 2 (UK representative sample) regressed on the self-reported real-world risky behaviours including exogeneous participant characteristics. Standard error in brackets. *** p < 0.001, ** p < 0.01, * p < 0.05.

|  |  |  |  |  |  |  |  |  |  |
| --- | --- | --- | --- | --- | --- | --- | --- | --- | --- |
| **BART** | **Model 1** | **Model 2** | **Model 3** | **Model 4** | **Model 5** | **Model 6** | **Model 7** | **Model 8** | **Model 9** |
|  |  |  |  |  |  |  |  |  |  |
|  |  |  |  |  |  |  |  |  |  |
| Intercept | 34.330*** | 31.520*** | 34.278*** | 27.750** | 40.335*** | 34.514*** | 33.482*** | 31.720*** | 32.187** |
|  | (8.640) | (8.680) | (8.664) | (8.830) | (9.220) | (8.677) | (8.810) | (9.337) | (12.085) |
| ln(Age) | 0.142 | 0.187 | 0.138 | 1.935 | 0.246 | 0.076 | 0.186 | 0.347 | 2.564 |
|  | (2.250) | (2.230) | (2.251) | (2.300) | (2.230) | (2.253) | (2.251) | (2.263) | (2.386) |
| Male | 1.407 | 1.103 | 1.321 | 0.288 | 1.353 | 1.440 | 1.353 | 1.362 | 0.065 |
|  | (1.710) | (1.690) | (1.709) | (1.750) | (1.690) | (1.707) | (1.702) | (1.694) | (1.786) |
| Asian | -0.989 | -0.334 | -1.351 | -1.288 | -0.415 | -1.358 | -1.367 | -1.209 | 0.293 |
|  | (2.890) | (2.860) | (2.875) | (3.030) | (2.870) | (2.842) | (2.840) | (2.836) | (3.199) |
| Black | -0.227 | 0.738 | -0.274 | -0.693 | 0.126 | -0.323 | 0.059 | -0.089 | 0.312 |
|  | (3.390) | (3.410) | (3.426) | (3.630) | (3.380) | (3.397) | (3.440) | (3.391) | (3.729) |
| Mixed or Multiple | -5.000 | -4.647 | -5.113 | -5.214 | -4.554 | -5.092 | -4.909 | -4.989 | -4.253 |
|  | (4.300) | (4.270) | (4.298) | (4.260) | (4.280) | (4.303) | (4.300) | (4.291) | (4.303) |
| Other Ethnic Group | 10.834 | 11.816 | 10.873 | 19.704 | 11.061 | 10.756 | 10.687 | 11.806 | 20.317 |
|  | (7.400) | (7.360) | (7.467) | (14.510) | (7.360) | (7.399) | (7.396) | (7.495) | (14.691) |
| Smoker | 0.001 |  |  |  |  |  |  |  | 1.234 |
|  | (2.490) |  |  |  |  |  |  |  | (2.628) |
| Drinker |  | 3.661 |  |  |  |  |  |  | 2.534 |
|  |  | (1.910) |  |  |  |  |  |  | (2.010) |
| Left House |  |  | 0.056 |  |  |  |  |  | -0.219 |
|  |  |  | (0.390) |  |  |  |  |  | (0.476) |
| Mask Ratio |  |  |  | 2.044 |  |  |  |  | 3.468 |
|  |  |  |  | (2.020) |  |  |  |  | (2.305) |
| Precautions |  |  |  |  | -1.800 |  |  |  | -2.433 |
|  |  |  |  |  | (1.080) |  |  |  | (1.408) |
| Contact Outside |  |  |  |  |  | 0.123 |  |  | -0.346 |
|  |  |  |  |  |  | (0.756) |  |  | (0.816) |
| Contact Inside |  |  |  |  |  |  | 0.220 |  | 0.181 |
|  |  |  |  |  |  |  | (0.439) |  | (0.475) |
| Isolation Status |  |  |  |  |  |  |  | 0.658 | -0.023 |
|  |  |  |  |  |  |  |  | (0.853) | (1.063) |
| Adj. R-sq | -0.009 | 0.004 | -0.009 | -0.006 | 0.001 | -0.008 | -0.008 | -0.007 | -0.011 |
| Obs. | 290 | 291 | 289 | 272 | 291 | 288 | 289 | 291 | 270 |
| F-Stat | 0.633 | 1.170 | 0.647 | 0.767 | 1.040 | 0.655 | 0.672 | 0.723 | 0.796 |
| p-value | 0.729 | 0.319 | 0.717 | 0.615 | 0.405 | 0.710 | 0.696 | 0.652 | 0.673 |

Table A6: Linear regression analysis – BEG for study 2 (UK representative sample) regressed on the self-reported real-world risky behaviours including exogeneous participant characteristics. Standard error in brackets. *** p < 0.001, ** p < 0.01, * p < 0.05.

|  |  |  |  |  |  |  |  |  |  |
| --- | --- | --- | --- | --- | --- | --- | --- | --- | --- |
| **BEG** | **Model 1** | **Model 2** | **Model 3** | **Model 4** | **Model 5** | **Model 6** | **Model 7** | **Model 8** | **Model 9** |
|  |  |  |  |  |  |  |  |  |  |
|  |  |  |  |  |  |  |  |  |  |
| Intercept | 5.102*** | 5.097*** | 4.963*** | 5.016*** | 6.133*** | 5.078*** | 5.153*** | 3.953*** | 5.166*** |
|  | (1.001) | (1.010) | (0.999) | (1.046) | (1.065) | (1.010) | (1.025) | (1.069) | (1.403) |
| ln(Age) | -0.518* | -0.528* | -0.562* | -0.462 | -0.510* | -0.515 | -0.524* | -0.419 | -0.351 |
|  | (0.260) | (0.259) | (0.260) | (0.273) | (0.257) | (0.262) | (0.261) | (0.259) | (0.281) |
| Male | 0.277 | 0.276 | 0.242 | 0.176 | 0.286 | 0.275 | 0.275 | 0.291 | 0.139 |
|  | (0.198) | (0.197) | (0.197) | (0.206) | (0.194) | (0.198) | (0.197) | (0.193) | (0.210) |
| Asian | -0.716* | -0.724* | -0.649 | -0.589 | -0.597 | -0.741* | -0.748* | -0.704* | -0.417 |
|  | (0.333) | (0.332) | (0.330) | (0.358) | (0.330) | (0.329) | (0.329) | (0.323) | (0.374) |
| Black | -0.001 | 0.013 | 0.095 | 0.187 | 0.052 | -0.017 | -0.014 | 0.052 | 0.243 |
|  | (0.392) | (0.395) | (0.394) | (0.428) | (0.388) | (0.393) | (0.398) | (0.386) | (0.436) |
| Mixed or Multiple | 0.323 | 0.332 | 0.329 | 0.308 | 0.400 | 0.316 | 0.320 | 0.331 | 0.404 |
|  | (0.496) | (0.495) | (0.494) | (0.502) | (0.491) | (0.498) | (0.497) | (0.488) | (0.503) |
| Other Ethnic Group | -1.069 | -1.038 | -0.840 | 1.465 | -1.019 | -1.050 | -1.060 | -0.637 | 1.783 |
|  | (0.853) | (0.854) | (0.858) | (1.709) | (0.844) | (0.856) | (0.855) | (0.853) | (1.715) |
| Smoker | 0.138 |  |  |  |  |  |  |  | 0.089 |
|  | (0.287) |  |  |  |  |  |  |  | (0.308) |
| Drinker |  | 0.082 |  |  |  |  |  |  | 0.067 |
|  |  | (0.220) |  |  |  |  |  |  | (0.234) |
| Left House |  |  | 0.084 |  |  |  |  |  | 0.014 |
|  |  |  | (0.045) |  |  |  |  |  | (0.056) |
| Mask Ratio |  |  |  | -0.102 |  |  |  |  | 0.186 |
|  |  |  |  | (0.240) |  |  |  |  | (0.271) |
| Precautions |  |  |  |  | -0.296* |  |  |  | -0.327* |
|  |  |  |  |  | (0.124) |  |  |  | (0.161) |
| Contact Outside |  |  |  |  |  | 0.039 |  |  | -0.080 |
|  |  |  |  |  |  | (0.088) |  |  | (0.096) |
| Contact Inside |  |  |  |  |  |  | -0.002 |  | -0.020 |
|  |  |  |  |  |  |  | (0.051) |  | (0.056) |
| Isolation Status |  |  |  |  |  |  |  | 0.278** | 0.185 |
|  |  |  |  |  |  |  |  | (0.097) | (0.124) |
| Adj. R-sq | 0.017 | 0.018 | 0.029 | 0.004 | 0.037 | 0.017 | 0.017 | 0.045 | 0.017 |
| Obs. | 288 | 289 | 287 | 270 | 289 | 286 | 287 | 289 | 268 |
| F-Stat | 1.700 | 1.750 | 2.200 | 1.170 | 2.580 | 1.690 | 1.690 | 2.940 | 1.330 |
| p-value | 0.108 | 0.097 | 0.034 | 0.320 | 0.014 | 0.110 | 0.111 | 0.005 | 0.190 |

Table A7: Linear regression analysis – DOSPERT for study 2 (UK representative sample) regressed on the self-reported real-world risky behaviours including exogeneous participant characteristics. Standard error in brackets. *** p < 0.001, ** p < 0.01, * p < 0.05.

|  |  |  |  |  |  |  |  |  |  |
| --- | --- | --- | --- | --- | --- | --- | --- | --- | --- |
| **DOSPERT** | **Model 1** | **Model 2** | **Model 3** | **Model 4** | **Model 5** | **Model 6** | **Model 7** | **Model 8** | **Model 9** |
|  |  |  |  |  |  |  |  |  |  |
|  |  |  |  |  |  |  |  |  |  |
| Intercept | 5.306*** | 5.206*** | 5.308*** | 5.433*** | 5.957*** | 5.313*** | 5.360*** | 5.174*** | 5.904*** |
|  | (0.445) | (0.449) | (0.449) | (0.464) | (0.473) | (0.451) | (0.458) | (0.485) | (0.600) |
| ln(Age) | -0.687*** | -0.701*** | -0.703*** | -0.707*** | -0.693*** | -0.688*** | -0.694*** | -0.685*** | -0.654*** |
|  | (0.116) | (0.115) | (0.117) | (0.121) | (0.115) | (0.117) | (0.117) | (0.118) | (0.121) |
| Male | 0.312*** | 0.307*** | 0.303*** | 0.315*** | 0.329*** | 0.317*** | 0.318*** | 0.327*** | 0.271** |
|  | (0.088) | (0.087) | (0.089) | (0.092) | (0.087) | (0.089) | (0.088) | (0.088) | (0.091) |
| Asian | -0.143 | -0.116 | -0.155 | -0.120 | -0.100 | -0.181 | -0.191 | -0.177 | 0.070 |
|  | (0.149) | (0.148) | (0.149) | (0.159) | (0.148) | (0.148) | (0.148) | (0.147) | (0.163) |
| Black | 0.067 | 0.120 | 0.083 | 0.062 | 0.087 | 0.040 | 0.055 | 0.064 | 0.144 |
|  | (0.175) | (0.176) | (0.177) | (0.191) | (0.174) | (0.176) | (0.179) | (0.176) | (0.189) |
| Mixed or Multiple | 0.165 | 0.196 | 0.170 | 0.154 | 0.215 | 0.160 | 0.169 | 0.172 | 0.218 |
|  | (0.221) | (0.221) | (0.223) | (0.224) | (0.221) | (0.223) | (0.224) | (0.223) | (0.219) |
| Other Ethnic Group | 0.170 | 0.265 | 0.270 | 0.577 | 0.219 | 0.206 | 0.190 | 0.281 | 0.610 |
|  | (0.381) | (0.381) | (0.387) | (0.762) | (0.379) | (0.384) | (0.385) | (0.390) | (0.746) |
| Smoker | 0.311* |  |  |  |  |  |  |  | 0.318* |
|  | (0.128) |  |  |  |  |  |  |  | (0.133) |
| Drinker |  | 0.264** |  |  |  |  |  |  | 0.233* |
|  |  | (0.098) |  |  |  |  |  |  | (0.102) |
| Left House |  |  | 0.027 |  |  |  |  |  | 0.002 |
|  |  |  | (0.020) |  |  |  |  |  | (0.024) |
| Mask Ratio |  |  |  | 0.021 |  |  |  |  | 0.122 |
|  |  |  |  | (0.106) |  |  |  |  | (0.117) |
| Precautions |  |  |  |  | -0.168** |  |  |  | -0.222** |
|  |  |  |  |  | (0.055) |  |  |  | (0.069) |
| Contact Outside |  |  |  |  |  | 0.046 |  |  | 0.020 |
|  |  |  |  |  |  | (0.039) |  |  | (0.041) |
| Contact Inside |  |  |  |  |  |  | 0.006 |  | -0.004 |
|  |  |  |  |  |  |  | (0.023) |  | (0.024) |
| Isolation Status |  |  |  |  |  |  |  | 0.058 | -0.046 |
|  |  |  |  |  |  |  |  | (0.044) | (0.054) |
| Adj. R-sq | 0.158 | 0.162 | 0.140 | 0.133 | 0.168 | 0.138 | 0.135 | 0.146 | 0.184 |
| Obs. | 291 | 292 | 290 | 273 | 292 | 289 | 290 | 292 | 271 |
| F-Stat | 8.790 | 9.040 | 7.730 | 6.980 | 9.410 | 7.580 | 7.470 | 8.100 | 5.360 |
| p-value | < .001 | < .001 | < .001 | < .001 | < .001 | < .001 | < .001 | < .001 | < .001 |

Table A8: Linear regression analysis – SOEP for study 2 (UK representative sample) regressed on the self-reported real-world risky behaviours including exogeneous participant characteristics. Standard error in brackets. *** p < 0.001, ** p < 0.01, * p < 0.05.

|  |  |  |  |  |  |  |  |  |  |
| --- | --- | --- | --- | --- | --- | --- | --- | --- | --- |
| **SOEP** | **Model 1** | **Model 2** | **Model 3** | **Model 4** | **Model 5** | **Model 6** | **Model 7** | **Model 8** | **Model 9** |
|  |  |  |  |  |  |  |  |  |  |
|  |  |  |  |  |  |  |  |  |  |
| Intercept | 9.478*** | 9.176*** | 9.258*** | 9.893*** | 10.342*** | 9.429*** | 9.765*** | 8.490*** | 9.189*** |
|  | (1.346) | (1.359) | (1.345) | (1.384) | (1.448) | (1.358) | (1.381) | (1.456) | (1.816) |
| ln(Age) | -1.314*** | -1.348*** | -1.361*** | -1.391*** | -1.349*** | -1.301*** | -1.335*** | -1.252*** | -1.314*** |
|  | (0.350) | (0.349) | (0.350) | (0.361) | (0.352) | (0.353) | (0.353) | (0.353) | (0.367) |
| Male | 1.207*** | 1.162*** | 1.117*** | 1.042*** | 1.215*** | 1.178*** | 1.182*** | 1.218*** | 0.971*** |
|  | (0.266) | (0.264) | (0.265) | (0.273) | (0.266) | (0.267) | (0.266) | (0.264) | (0.274) |
| Asian | 0.542 | 0.557 | 0.521 | 0.494 | 0.468 | 0.386 | 0.371 | 0.416 | 0.993* |
|  | (0.450) | (0.448) | (0.446) | (0.475) | (0.453) | (0.445) | (0.445) | (0.442) | (0.492) |
| Black | 0.838 | 0.992 | 0.966 | 0.958 | 0.849 | 0.781 | 0.755 | 0.872 | 1.135* |
|  | (0.529) | (0.533) | (0.532) | (0.569) | (0.533) | (0.532) | (0.539) | (0.529) | (0.573) |
| Mixed or Multiple | -0.724 | -0.649 | -0.714 | -0.763 | -0.666 | -0.743 | -0.743 | -0.709 | -0.686 |
|  | (0.670) | (0.669) | (0.668) | (0.668) | (0.675) | (0.674) | (0.674) | (0.670) | (0.662) |
| Other Ethnic Group | -0.142 | 0.079 | 0.250 | 0.519 | -0.082 | -0.074 | -0.088 | 0.332 | 0.704 |
|  | (1.154) | (1.154) | (1.160) | (2.274) | (1.160) | (1.158) | (1.160) | (1.170) | (2.260) |
| Smoker | 0.537 |  |  |  |  |  |  |  | 0.447 |
|  | (0.388) |  |  |  |  |  |  |  | (0.404) |
| Drinker |  | 0.697* |  |  |  |  |  |  | 0.573 |
|  |  | (0.298) |  |  |  |  |  |  | (0.308) |
| Left House |  |  | 0.130* |  |  |  |  |  | 0.101 |
|  |  |  | (0.060) |  |  |  |  |  | (0.073) |
| Mask Ratio |  |  |  | 0.228 |  |  |  |  | 0.414 |
|  |  |  |  | (0.317) |  |  |  |  | (0.355) |
| Precautions |  |  |  |  | -0.187 |  |  |  | -0.183 |
|  |  |  |  |  | (0.168) |  |  |  | (0.209) |
| Contact Outside |  |  |  |  |  | 0.115 |  |  | -0.003 |
|  |  |  |  |  |  | (0.118) |  |  | (0.125) |
| Contact Inside |  |  |  |  |  |  | -0.032 |  | -0.094 |
|  |  |  |  |  |  |  | (0.069) |  | (0.072) |
| Isolation Status |  |  |  |  |  |  |  | 0.292* | 0.146 |
|  |  |  |  |  |  |  |  | (0.133) | (0.162) |
| Adj. R-sq | 0.118 | 0.124 | 0.115 | 0.108 | 0.111 | 0.103 | 0.102 | 0.122 | 0.132 |
| Obs. | 291 | 292 | 290 | 273 | 292 | 289 | 290 | 292 | 271 |
| F-Stat | 6.560 | 6.920 | 6.390 | 5.710 | 6.220 | 5.730 | 5.710 | 6.810 | 3.940 |
| p-value | < .001 | < .001 | < .001 | < .001 | < .001 | < .001 | < .001 | < .001 | < .001 |

**Supplementary Materials B: Participant Demographics**

Table B1: Demographic description of the participants for Study 1 and Study 2.

|  |  |  |  |  |  |
| --- | --- | --- | --- | --- | --- |
|  |  | **Study 1** | | **Study 2** | |
|  |  |  |  |  |  |
| **Age (years)** | Mean | 32.56 | | 45.57 | |
|  | SD | 12.05 | | 15.79 | |
|  | Min | 18.00 | | 18.00 | |
|  | Max | 77.00 | | 75.00 | |
|  |  |  |  |  |  |
|  |  | **Count** | **Percent** | **Count** | **Percent** |
|  |  |  |  |  |  |
| **Gender** | Male | 308 | 32.73% | 146 | 49.16% |
|  | Female | 633 | 67.27% | 151 | 50.84% |
| **Ethnicity** | White | 813 | 85.76% | 228 | 77.55% |
|  | Black | 25 | 2.64% | 20 | 6.80% |
|  | Asian | 70 | 7.38% | 30 | 10.20% |
|  | Other Ethnic Group | 12 | 1.27% | 4 | 1.36% |
|  | Mixed or Multiple ethnic groups | 28 | 2.95% | 12 | 4.08% |
| **Education** | Primary school | 3 | 0.32% | 1 | 0.34% |
|  | Secondary school | 382 | 40.21% | 131 | 44.26% |
|  | Higher Education | 565 | 59.47% | 164 | 55.41% |

**Supplementary Materials C: Adapted BEG Task**

*Screenshot of the instructions:*

**
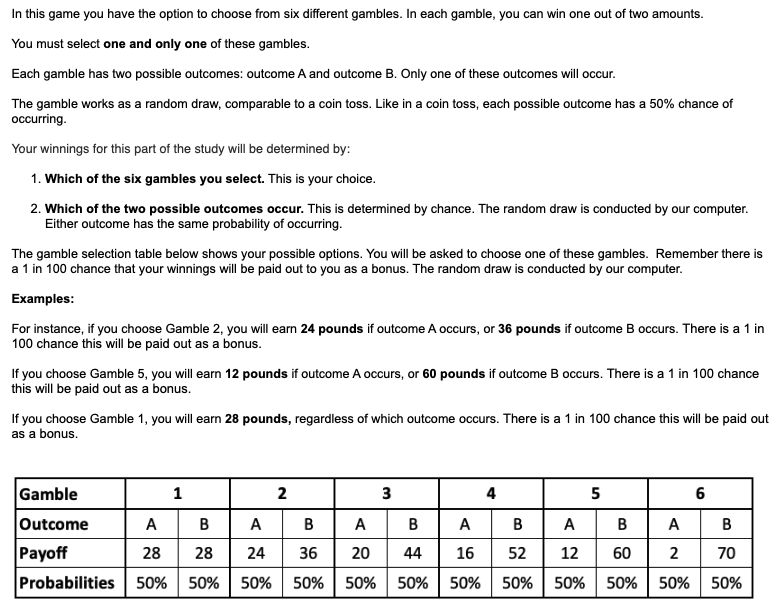
**

*Screenshot of the participant choice screen:*

**
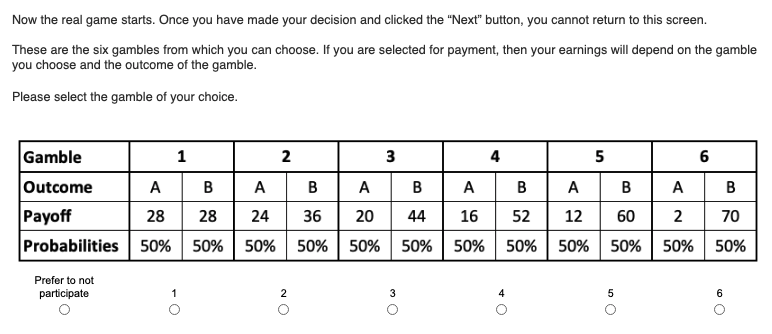
**

**Supplementary Materials D: Precautions index items**

Please answer the below questions. As a reminder, please exclude any measures that you have already taken for reasons other than coronavirus (COVID-19).

Thinking about the last 7 days…

| Not at all | Rarely | Sometimes | Frequently | Always |
| --- | --- | --- | --- | --- |

1. how often have you avoided contact with people who have symptoms or you think may have been exposed to the coronavirus to protect yourself or others from coronavirus (COVID-19)?
2. how often have you avoided crowded areas to protect yourself or others from coronavirus (COVID-19)?
3. how often have you avoided going out in general to protect yourself or others from coronavirus (COVID-19)?
4. how often have you avoided going to hospital or other healthcare settings to protect yourself or others from coronavirus (COVID-19)?
5. how often have you avoided going to shops to protect yourself or others from coronavirus (COVID-19)?
6. how often have you avoided having guests to your home to protect yourself or others from coronavirus (COVID-19)?
7. how often have you avoided large-sized social gatherings (more than 10 people) to protect yourself or others from coronavirus (COVID-19)?
8. how often have you avoided letting your children go to school/ university (if you have children) to protect yourself or others from coronavirus (COVID-19)?
9. how often have you avoided medium-sized social gatherings (between 3 and 10 people) to protect yourself or others from coronavirus (COVID-19)?
10. how often have you avoided small social gatherings (not more than 2 people) to protect yourself or others from coronavirus (COVID-19)?
11. how often have you avoided taking public transport to protect yourself or others from coronavirus (COVID-19)?
12. how often have you avoided touching objects in public (e.g. elevator buttons or doors) to protect yourself or others from coronavirus (COVID-19)?
13. how often have you avoided working outside your home (if job is full or part-time) to protect yourself or others from coronavirus (COVID-19)?
14. how often have you cleaned frequently touched surfaces in the home (e.g. doorknobs, toilets, taps) to protect yourself or others from coronavirus (COVID-19)?
15. how often have you covered your nose and mouth when sneezing or coughing to protect yourself or others from coronavirus (COVID-19)?
16. how often have you eaten separately at home, when normally you would eat a meal with others, to protect yourself or others from coronavirus (COVID-19)?
17. how often have you slept in separate bedrooms at home, when normally you would share a bedroom to protect yourself or others from coronavirus (COVID-19)?
18. how often have you used hand sanitiser to protect yourself or others from coronavirus (COVID-19)?
19. how often have you washed hands with soap and water to protect yourself or others from coronavirus (COVID-19)?
20. how often have you worn a face mask outside your home to protect yourself or others from coronavirus (COVID-19)?

**Supplementary Materials E: Mask Compensation Behaviour index items**

1. On the days when you wore a mask, did you leave your home more, less or about the same?
2. On the days when you wore a mask, did you touch objects in public (like elevator buttons or doors) more, less or about the same?
3. On the days when you wore a mask, did you wash your hands more, less or about the same? (Reverse scoring)
4. On the days when you wore a mask, were you around people with coronavirus symptoms more, less or about the same?
5. On the days when you wore a mask, were you around vulnerable people with other health conditions more, less or about the same?
6. On the days when you wore a mask, were you in close physical contact with people more, less or about the same?

**Supplementary Materials F: Forest plot for DOSPERT and SOEP domains**

Figure F1: Forest plot for the standardised (z-scored) mean differences with 95% confidence interval across the specific risk measure domains in study 1 (general sample) and study 2 (UK representative sample) by gender, age (classified as young and old by median age) and self-reported health (classified as more and less healthy by median self-reported health). A positive number indicates higher risk tolerance by male participants compared to female participants, by younger compared to older participants, and by healthier compared to less healthy participants respectively. The DOSPERT domains are: social (S), recreational (R), financial (F), health/safety (H/S), and ethical (E). The SOEP domains are: health (H), financial (F), career (C), driving (D), and leisure and sport (L/S).

**
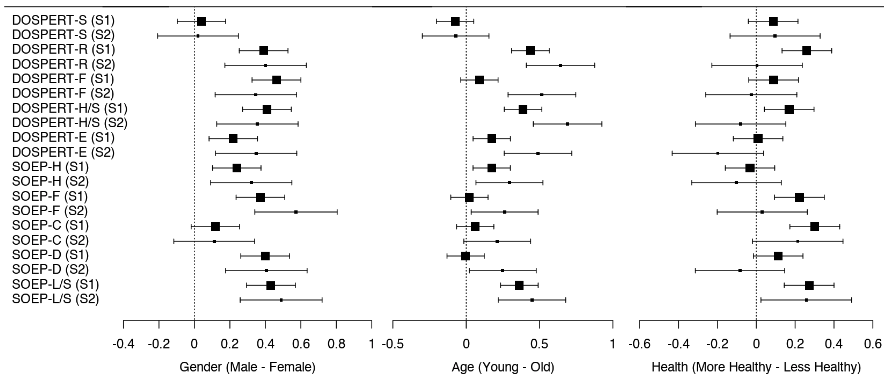
**

**Supplementary Materials G: Robustness analyses for risk-taking measures as predictors**

Table G1: Linear regression analysis – self-reported real-world risky behaviours for study 2 (UK representative sample) regressed on the BART and exogenous participant characteristics. Standard error in brackets. *** p < 0.001, ** p < 0.01, * p < 0.05.

|  |  |  |  |  |  |  |  |  |
| --- | --- | --- | --- | --- | --- | --- | --- | --- |
|  | **Smoker** | **Drinker** | **Left House** | **Mask Ratio** | **Precautions** | **Contact Outside** | **Contact Inside** | **Isolation Status** |
|  |  |  |  |  |  |  |  |  |
|  |  |  |  |  |  |  |  |  |
| Intercept | 0.311 | 0.711** | 2.587 | 0.454 | 3.389*** | 1.389* | 4.217*** | 4.219*** |
|  | (0.211) | (0.271) | (1.350) | (0.271) | (0.478) | (0.699) | (1.198) | (0.612) |
| ln(Age) | -0.053 | -0.027 | 0.329 | -0.062 | 0.089 | -0.133 | -0.287 | -0.394* |
|  | (0.054) | (0.069) | (0.343) | (0.070) | (0.121) | (0.178) | (0.305) | (0.155) |
| Male | 0.061 | 0.061 | 0.395 | 0.042 | 0.013 | -0.081 | -0.068 | -0.034 |
|  | (0.041) | (0.052) | (0.260) | (0.053) | (0.092) | (0.135) | (0.231) | (0.118) |
| Asian | -0.047 | -0.262** | -1.157** | 0.337*** | 0.489** | -0.126 | 0.288 | -0.146 |
|  | (0.069) | (0.087) | (0.434) | (0.090) | (0.154) | (0.224) | (0.385) | (0.197) |
| Black | -0.049 | -0.264* | -1.238* | 0.392*** | 0.198 | 0.212 | -1.322** | -0.218 |
|  | (0.081) | (0.104) | (0.518) | (0.108) | (0.184) | (0.268) | (0.459) | (0.235) |
| Mixed or Multiple | 0.020 | -0.080 | -0.042 | 0.046 | 0.223 | 0.203 | -0.411 | -0.007 |
|  | (0.103) | (0.132) | (0.658) | (0.130) | (0.233) | (0.340) | (0.584) | (0.299) |
| Other Ethnic Group | 0.081 | -0.313 | -2.677* | 0.606 | 0.198 | -0.197 | 0.574 | -1.553** |
|  | (0.177) | (0.228) | (1.133) | (0.440) | (0.402) | (0.586) | (1.005) | (0.515) |
| BART | 0.000 | 0.004 | 0.001 | 0.002 | -0.005 | 0.001 | 0.004 | 0.003 |
|  | (0.001) | (0.002) | (0.009) | (0.002) | (0.003) | (0.005) | (0.008) | (0.004) |
| Adj. R-sq | -0.009 | 0.045 | 0.048 | 0.083 | 0.025 | -0.016 | 0.015 | 0.025 |
| Obs. | 290 | 291 | 289 | 272 | 291 | 288 | 289 | 291 |
| F-Stat | 0.616 | 2.940 | 3.070 | 4.490 | 2.060 | 0.343 | 1.650 | 2.060 |
| p-value | 0.743 | 0.006 | 0.004 | < .001 | 0.048 | 0.933 | 0.123 | 0.048 |

Table G2: Linear regression analysis – self-reported real-world risky behaviours for study 2 (UK representative sample) regressed on the BEG and exogenous participant characteristics. Standard error in brackets. *** p < 0.001, ** p < 0.01, * p < 0.05.

|  |  |  |  |  |  |  |  |  |
| --- | --- | --- | --- | --- | --- | --- | --- | --- |
|  | **Smoker** | **Drinker** | **Left House** | **Mask Ratio** | **Precautions** | **Contact Outside** | **Contact Inside** | **Isolation Status** |
|  |  |  |  |  |  |  |  |  |
|  |  |  |  |  |  |  |  |  |
| Intercept | 0.290 | 0.774** | 1.560 | 0.527 | 3.618*** | 1.382 | 4.469*** | 3.830*** |
|  | (0.217) | (0.281) | (1.368) | (0.279) | (0.491) | (0.716) | (1.223) | (0.619) |
| ln(Age) | -0.052 | -0.019 | 0.479 | -0.059 | 0.032 | -0.139 | -0.313 | -0.346* |
|  | (0.054) | (0.071) | (0.344) | (0.070) | (0.123) | (0.180) | (0.307) | (0.155) |
| Male | 0.062 | 0.067 | 0.401 | 0.046 | 0.033 | -0.074 | -0.052 | -0.062 |
|  | (0.041) | (0.053) | (0.259) | (0.053) | (0.093) | (0.136) | (0.232) | (0.117) |
| Asian | -0.044 | -0.255** | -1.016* | 0.337*** | 0.450** | -0.124 | 0.269 | -0.075 |
|  | (0.070) | (0.089) | (0.433) | (0.090) | (0.156) | (0.227) | (0.387) | (0.196) |
| Black | -0.051 | -0.258* | -1.205* | 0.399*** | 0.203 | 0.202 | -1.333** | -0.218 |
|  | (0.081) | (0.106) | (0.513) | (0.108) | (0.184) | (0.268) | (0.458) | (0.232) |
| Mixed or Multiple | 0.016 | -0.093 | -0.065 | 0.044 | 0.277 | 0.183 | -0.439 | -0.056 |
|  | (0.103) | (0.134) | (0.650) | (0.129) | (0.233) | (0.340) | (0.581) | (0.294) |
| Other Ethnic Group | 0.085 | -0.260 | -2.449* | 0.660 | 0.066 | -0.186 | 0.595 | -1.413** |
|  | (0.178) | (0.231) | (1.121) | (0.439) | (0.402) | (0.586) | (1.002) | (0.508) |
| BEG | 0.006 | 0.006 | 0.144 | -0.007 | -0.067* | 0.018 | -0.003 | 0.101** |
|  | (0.012) | (0.016) | (0.078) | (0.016) | (0.028) | (0.041) | (0.070) | (0.035) |
| Adj. R-sq | -0.008 | 0.030 | 0.061 | 0.083 | 0.035 | -0.016 | 0.016 | 0.051 |
| Obs. | 288 | 289 | 287 | 270 | 289 | 286 | 287 | 289 |
| F-Stat | 0.685 | 2.270 | 3.670 | 4.490 | 2.500 | 0.368 | 1.640 | 3.200 |
| p-value | 0.685 | 0.029 | < .001 | < .001 | 0.017 | 0.921 | 0.123 | 0.003 |

Table G3: Linear regression analysis – self-reported real-world risky behaviours for study 2 (UK representative sample) regressed on the DOSPERT and exogenous participant characteristics. Standard error in brackets. *** p < 0.001, ** p < 0.01, * p < 0.05.

|  |  |  |  |  |  |  |  |  |
| --- | --- | --- | --- | --- | --- | --- | --- | --- |
|  | **Smoker** | **Drinker** | **Left House** | **Mask Ratio** | **Precautions** | **Contact Outside** | **Contact Inside** | **Isolation Status** |
|  |  |  |  |  |  |  |  |  |
|  |  |  |  |  |  |  |  |  |
| Intercept | -0.044 | 0.317 | 1.326 | 0.467 | 4.200*** | 0.838 | 4.087** | 3.764*** |
|  | (0.250) | (0.324) | (1.611) | (0.329) | (0.575) | (0.833) | (1.438) | (0.732) |
| ln(Age) | -0.007 | 0.040 | 0.497 | -0.053 | -0.043 | -0.059 | -0.253 | -0.320 |
|  | (0.056) | (0.073) | (0.363) | (0.074) | (0.129) | (0.188) | (0.324) | (0.165) |
| Male | 0.040 | 0.040 | 0.341 | 0.043 | 0.083 | -0.107 | -0.054 | -0.057 |
|  | (0.041) | (0.053) | (0.265) | (0.054) | (0.094) | (0.137) | (0.236) | (0.120) |
| Asian | -0.036 | -0.246** | -1.102* | 0.338*** | 0.472** | -0.103 | 0.306 | -0.128 |
|  | (0.068) | (0.087) | (0.434) | (0.090) | (0.155) | (0.224) | (0.387) | (0.197) |
| Black | -0.051 | -0.267* | -1.238* | 0.393*** | 0.220 | 0.210 | -1.310** | -0.220 |
|  | (0.080) | (0.104) | (0.517) | (0.108) | (0.184) | (0.267) | (0.461) | (0.235) |
| Mixed or Multiple | 0.009 | -0.110 | -0.076 | 0.037 | 0.293 | 0.185 | -0.423 | -0.037 |
|  | (0.102) | (0.132) | (0.655) | (0.130) | (0.234) | (0.339) | (0.585) | (0.298) |
| Other Ethnic Group | 0.069 | -0.290 | -2.696* | 0.642 | 0.189 | -0.205 | 0.626 | -1.534** |
|  | (0.175) | (0.226) | (1.127) | (0.439) | (0.402) | (0.583) | (1.005) | (0.512) |
| DOSPERT | 0.065* | 0.093** | 0.236 | 0.007 | -0.189** | 0.106 | 0.043 | 0.102 |
|  | (0.027) | (0.035) | (0.174) | (0.035) | (0.062) | (0.090) | (0.155) | (0.079) |
| Adj. R-sq | 0.011 | 0.055 | 0.053 | 0.080 | 0.048 | -0.012 | 0.014 | 0.028 |
| Obs. | 291 | 292 | 290 | 273 | 292 | 289 | 290 | 292 |
| F-Stat | 1.480 | 3.430 | 3.330 | 4.390 | 3.090 | 0.531 | 1.590 | 2.210 |
| p-value | 0.174 | 0.002 | 0.002 | < .001 | 0.004 | 0.811 | 0.139 | 0.034 |

Table G4: Linear regression analysis – self-reported real-world risky behaviours for study 2 (UK representative sample) regressed on the SOEP and exogenous participant characteristics. Standard error in brackets. *** p < 0.001, ** p < 0.01, * p < 0.05.

|  |  |  |  |  |  |  |  |  |
| --- | --- | --- | --- | --- | --- | --- | --- | --- |
|  | **Smoker** | **Drinker** | **Left House** | **Mask Ratio** | **Precautions** | **Contact Outside** | **Contact Inside** | **Isolation Status** |
|  |  |  |  |  |  |  |  |  |
|  |  |  |  |  |  |  |  |  |
| Intercept | 0.189 | 0.559 | 1.409 | 0.419 | 3.399*** | 1.125 | 4.544*** | 3.764*** |
|  | (0.222) | (0.287) | (1.416) | (0.291) | (0.515) | (0.736) | (1.270) | (0.643) |
| ln(Age) | -0.036 | 0.011 | 0.496 | -0.046 | 0.060 | -0.093 | -0.314 | -0.315* |
|  | (0.055) | (0.071) | (0.349) | (0.072) | (0.127) | (0.182) | (0.313) | (0.158) |
| Male | 0.047 | 0.038 | 0.271 | 0.036 | 0.050 | -0.108 | -0.012 | -0.093 |
|  | (0.042) | (0.054) | (0.266) | (0.054) | (0.097) | (0.139) | (0.239) | (0.121) |
| Asian | -0.052 | -0.273** | -1.193** | 0.333*** | 0.516** | -0.134 | 0.306 | -0.168 |
|  | (0.069) | (0.087) | (0.431) | (0.090) | (0.157) | (0.224) | (0.386) | (0.195) |
| Black | -0.058 | -0.284** | -1.326* | 0.385*** | 0.229 | 0.192 | -1.289** | -0.261 |
|  | (0.081) | (0.105) | (0.516) | (0.108) | (0.188) | (0.268) | (0.463) | (0.234) |
| Mixed or Multiple | 0.029 | -0.075 | 0.053 | 0.044 | 0.244 | 0.224 | -0.433 | 0.022 |
|  | (0.102) | (0.132) | (0.653) | (0.130) | (0.237) | (0.339) | (0.585) | (0.296) |
| Other Ethnic Group | 0.083 | -0.269 | -2.638* | 0.640 | 0.150 | -0.182 | 0.632 | -1.508** |
|  | (0.176) | (0.227) | (1.121) | (0.438) | (0.408) | (0.583) | (1.005) | (0.509) |
| SOEP | 0.013 | 0.027* | 0.124* | 0.009 | -0.023 | 0.029 | -0.024 | 0.057* |
|  | (0.009) | (0.012) | (0.058) | (0.012) | (0.021) | (0.030) | (0.052) | (0.026) |
| Adj. R-sq | -0.002 | 0.050 | 0.063 | 0.082 | 0.021 | -0.013 | 0.014 | 0.039 |
| Obs. | 291 | 292 | 290 | 273 | 292 | 289 | 290 | 292 |
| F-Stat | 0.903 | 3.170 | 3.760 | 4.460 | 1.880 | 0.470 | 1.610 | 2.670 |
| p-value | 0.504 | 0.003 | < .001 | < .001 | 0.073 | 0.856 | 0.133 | 0.011 |

Table G5: Linear regression analysis – self-reported real-world risky behaviours for study 2 (UK representative sample) regressed on the risk measures and exogenous participant characteristics. Standard error in brackets. *** p < 0.001, ** p < 0.01, * p < 0.05.

|  |  |  |  |  |  |  |  |  |
| --- | --- | --- | --- | --- | --- | --- | --- | --- |
|  | **Smoker** | **Drinker** | **Left House** | **Mask Ratio** | **Precautions** | **Contact Outside** | **Contact Inside** | **Isolation Status** |
|  |  |  |  |  |  |  |  |  |
|  |  |  |  |  |  |  |  |  |
| Intercept | -0.034 | 0.250 | 0.765 | 0.418 | 4.418*** | 0.851 | 4.370** | 3.467*** |
|  | (0.257) | (0.330) | (1.627) | (0.336) | (0.572) | (0.856) | (1.459) | (0.738) |
| ln(Age) | -0.007 | 0.039 | 0.585 | -0.053 | -0.055 | -0.068 | -0.324 | -0.300 |
|  | (0.057) | (0.073) | (0.362) | (0.075) | (0.127) | (0.191) | (0.325) | (0.164) |
| Male | 0.038 | 0.020 | 0.268 | 0.035 | 0.051 | -0.124 | -0.064 | -0.121 |
|  | (0.043) | (0.054) | (0.268) | (0.055) | (0.094) | (0.141) | (0.241) | (0.122) |
| Asian | -0.039 | -0.261** | -1.087* | 0.326*** | 0.412** | -0.128 | 0.276 | -0.109 |
|  | (0.070) | (0.088) | (0.436) | (0.091) | (0.153) | (0.230) | (0.391) | (0.198) |
| Black | -0.057 | -0.280** | -1.301* | 0.386*** | 0.188 | 0.178 | -1.331** | -0.261 |
|  | (0.081) | (0.104) | (0.515) | (0.109) | (0.181) | (0.271) | (0.461) | (0.233) |
| Mixed or Multiple | 0.004 | -0.073 | 0.010 | 0.063 | 0.281 | 0.182 | -0.450 | -0.012 |
|  | (0.104) | (0.133) | (0.656) | (0.131) | (0.231) | (0.345) | (0.588) | (0.298) |
| Other Ethnic Group | 0.074 | -0.321 | -2.501* | 0.622 | 0.150 | -0.215 | 0.521 | -1.445** |
|  | (0.178) | (0.228) | (1.125) | (0.442) | (0.395) | (0.592) | (1.009) | (0.510) |
| BART | -0.001 | 0.003 | 0.002 | 0.002 | -0.004 | 0.000 | 0.005 | 0.002 |
|  | (0.002) | (0.002) | (0.009) | (0.002) | (0.003) | (0.005) | (0.009) | (0.004) |
| BEG | 0.002 | -0.007 | 0.121 | -0.012 | -0.048 | 0.010 | 0.001 | 0.088* |
|  | (0.013) | (0.017) | (0.082) | (0.017) | (0.029) | (0.043) | (0.073) | (0.037) |
| DOSPERT | 0.065* | 0.057 | -0.010 | -0.006 | -0.164* | 0.077 | 0.027 | -0.014 |
|  | (0.032) | (0.041) | (0.204) | (0.042) | (0.071) | (0.107) | (0.183) | (0.092) |
| SOEP | 0.002 | 0.019 | 0.101 | 0.011 | 0.016 | 0.018 | -0.021 | 0.048 |
|  | (0.011) | (0.014) | (0.067) | (0.014) | (0.023) | (0.035) | (0.060) | (0.030) |
| Adj. R-sq | 0.002 | 0.058 | 0.062 | 0.077 | 0.050 | -0.021 | 0.008 | 0.053 |
| Obs. | 287 | 288 | 286 | 269 | 288 | 285 | 286 | 288 |
| F-Stat | 1.070 | 2.780 | 2.900 | 3.240 | 2.510 | 0.417 | 1.220 | 2.600 |
| p-value | 0.385 | 0.003 | 0.002 | < .001 | 0.007 | 0.938 | 0.276 | 0.005 |

**Supplementary Materials H: Health specific risk-taking measures as predictors of health and Covid-19 risky behaviours**

Table H1: Linear regression analysis – self-reported real-world risky behaviours for study 2 (UK representative sample) regressed on the health specific scales for the DOSPERT and SOEP. Standard error in brackets. *** p < 0.001, ** p < 0.01, * p < 0.05.

|  |  |  |  |  |  |  |  |  |  |
| --- | --- | --- | --- | --- | --- | --- | --- | --- | --- |
|  |  | **Smoker** | **Drinker** | **Left House** | **Mask Ratio** | **Precautions** | **Contact Outside** | **Contact Inside** | **Isolation Status** |
|  |  |  |  |  |  |  |  |  |  |
|  |  |  |  |  |  |  |  |  |  |
| Intercept |  | 0.430*** | -0.013 | 3.142*** | 0.480*** | 4.054*** | 0.799*** | 2.549*** | 2.508*** |
|  |  | (0.069) | (0.052) | (0.352) | (0.074) | (0.123) | (0.176) | (0.309) | (0.156) |
| DOSPERT_H/S | | 0.095*** | 0.053** | 0.242* | -0.037 | -0.157*** | 0.029 | 0.216* | 0.097 |
|  |  | (0.023) | (0.017) | (0.116) | (0.024) | (0.041) | (0.058) | (0.102) | (0.052) |
| Adj. R-sq |  | 0.051 | 0.027 | 0.011 | 0.005 | 0.045 | -0.003 | 0.012 | 0.008 |
| Obs. |  | 298 | 297 | 295 | 278 | 297 | 294 | 295 | 298 |
| F-Stat |  | 17.100 | 9.220 | 4.320 | 2.360 | 15.000 | 0.252 | 4.450 | 3.540 |
| p-value |  | < .001 | 0.003 | 0.038 | 0.126 | < .001 | 0.616 | 0.036 | 0.061 |
|  |  |  |  |  |  |  |  |  |  |
| Intercept |  | 0.632*** | 0.063* | 3.436*** | 0.455*** | 3.799*** | 0.856*** | 3.131*** | 2.530*** |
|  |  | (0.041) | (0.030) | (0.201) | (0.042) | (0.070) | (0.101) | (0.178) | (0.088) |
| SOEP_H |  | 0.023* | 0.025** | 0.135* | -0.027* | -0.066*** | 0.009 | 0.009 | 0.088*** |
|  |  | (0.011) | (0.008) | (0.054) | (0.011) | (0.019) | (0.027) | (0.048) | (0.024) |
| Adj. R-sq |  | 0.011 | 0.029 | 0.018 | 0.018 | 0.036 | -0.003 | -0.003 | 0.042 |
| Obs. |  | 298 | 297 | 295 | 278 | 297 | 294 | 295 | 298 |
| F-Stat |  | 4.310 | 9.920 | 6.360 | 6.040 | 12.100 | 0.109 | 0.033 | 14.200 |
| p-value |  | 0.039 | 0.002 | 0.012 | 0.015 | < .001 | 0.741 | 0.855 | < .001 |

**Supplementary Materials I: Mask Compensation Behaviour**

Table I1: Linear regression analysis – Mask Compensation Behaviour for study 2 (UK representative sample) regressed on exogeneous participant characteristics. Standard error in brackets. *** p < 0.001, ** p < 0.01, * p < 0.05.

|  |  |
| --- | --- |
|  | **Mask Compensation Behaviour** |
|  |  |
|  |  |
| Intercept | -0.175 |
|  | (0.206) |
| ln(Age) | 0.014 |
|  | (0.054) |
| Male | -0.023 |
|  | (0.040) |
| Black | -0.140* |
|  | (0.065) |
| Mixed or Multiple | -0.029 |
|  | (0.103) |
| Asian | -0.231** |
|  | (0.077) |
| Other Ethnic Group | -0.059 |
|  | (0.162) |
| Adj. R-sq | 0.029 |
| Obs. | 246 |
| F-Stat | 2.240 |
| p-value | 0.040 |
